# *BCL2A1*^high^ CD8^+^ T Cells Are a Survival-Associated Predictor of Immune Checkpoint Blockade Response in Lung Adenocarcinoma

**DOI:** 10.3390/diagnostics16030475

**Published:** 2026-02-03

**Authors:** Hoang Minh Quan Pham, Po-Hao Feng, Chia-Ling Chen, Kang-Yun Lee, Chiou-Feng Lin

**Affiliations:** 1 International Ph.D. Program in Medicine, College of Medicine, Taipei Medical University, Taipei 110, Taiwan; d142112005@tmu.edu.tw; 2 Department of Oncology, Faculty of Medicine, Can Tho University of Medicine and Pharmacy, Can Tho 94000, Vietnam; 3 Department of Microbiology and Immunology, School of Medicine, College of Medicine, Taipei Medical University, Taipei 110, Taiwan; 4 Division of Pulmonary Medicine, Department of Internal Medicine, Shuang Ho Hospital, Taipei Medical University, New Taipei City 235, Taiwan; fengpohao@tmu.edu.tw (P.-H.F.); leekangyun@tmu.edu.tw (K.-Y.L.); 5 Division of Pulmonary Medicine, Department of Internal Medicine, School of Medicine, College of Medicine, Taipei Medical University, Taipei 110, Taiwan; 6 School of Respiratory Therapy, College of Medicine, Taipei Medical University, Taipei 110, Taiwan; chialing66@tmu.edu.tw; 7 Graduate Institute of Clinical Medicine, College of Medicine, Taipei Medical University, Taipei 110, Taiwan; 8 TMU Research Center of Thoracic Medicine, Taipei Medical University, Taipei 110, Taiwan; 9 Graduate Institute of Medical Sciences, College of Medicine, Taipei Medical University, Taipei 110, Taiwan; 10 Core Laboratory of Immune Monitoring, Office of Research & Development, Taipei Medical University, Taipei 110, Taiwan

**Keywords:** lung adenocarcinoma, immune checkpoint blockade, *BCL2A1*, CD8^+^ T cells, single-cell RNA-seq

## Abstract

**Background**: Immune checkpoint blockade (ICB) has revolutionized lung adenocarcinoma (LUAD) therapy, yet predictive bio-markers remain suboptimal. We hypothesized that *BCL2A1* expression in CD8^+^ T cells may reflect immune endurance and complement PD-L1 in predicting ICB response. **Methods**: Integrating bulk and single-cell RNA-seq across multiple LUAD cohorts, this study performed differential expression, survival, and pathway analyses in a discovery cohort (*n* = 60) and validated findings across five independent cohorts (*n* = 126). **Results**: Single-cell profiling identified *BCL2A1* enrichment in tissue-resident memory and proliferating subsets that appeared preferentially expanded in responders; cell–cell communication analysis revealed that *BCL2A1*^high^ CD8^+^ T cells exhibited significantly enhanced outgoing signaling capacity (*p* = 0.0278), with proliferating subsets serving as intra-CD8^+^ coordination hubs and MIF pathway interactions achieving the highest intensity among all axes examined. *BCL2A1* was significantly upregulated in responders (FDR < 0.05) and associated with improved ICB survival (HR = 0.43, *p* < 0.05), but not in non-ICB settings, suggesting treatment-specific prognostic relevance. A tri-marker model integrating *BCL2A1*, *PD-L1* (CD274), and a 27-gene HOT score demonstrated favorable predictive performance (AUC = 0.826 discovery; macro-AUC = 0.774 validation), outperforming *PD-L1* alone (AUC = 0.706) and established signatures including TIDE, IPS, TIS, and IFNG. Cross-platform simulations suggested high reproducibility (ρ = 0.982–0.993). **Conclusions**: These findings suggest *BCL2A1* may serve as a bio-marker of CD8^+^ T-cell survival and enhanced intercellular coordination, and its integration with PD-L1 and immune activation markers may yield a reproducible ICB response predictor, pending clinical validation.

## 1. Introduction

Immune checkpoint blockade (ICB) has revolutionized the treatment of advanced lung adenocarcinoma (LUAD), yet only a subset of patients receives durable clinical benefit [1,2,3]. This variability underscores a critical unmet need for reliable bio-markers to optimize patient selection, thereby maximizing efficacy while minimizing unnecessary toxicity and cost [3,4]. The current clinical standard, programmed death-ligand 1 (PD-L1, encoded by CD274), offers only modest predictive accuracy [3,5]. Its limitations are well-documented: many PD-L1-negative patients still respond to ICB, while a significant portion of PD-L1-positive patients do not [4]. Furthermore, as a static marker, PD-L1 expression fails to capture the dynamic functional states of CD8^+^ T cells, the primary effectors of anti-tumor immunity, and is subject to spatial and temporal heterogeneity [1].

These shortcomings suggest that relying on a single biological axis is insufficient to encapsulate the complexity of the tumor–immune microenvironment. Consequently, there is a pressing need to identify novel bio-markers that provide orthogonal, complementary information to PD-L1. *BCL2A1* (B-cell lymphoma 2-related protein A1), an anti-apoptotic gene from the BCL-2 family, is known to regulate the survival of immune cells, including CD8^+^ T cells [6,7]. It is hypothesized that *BCL2A1* expression could serve as a functional bio-marker reflecting the survival capacity and fitness of effector T cells within the tumor microenvironment. A robust and sustained anti-tumor immune response requires not only T-cell activation but also their persistence in a hostile environment. Therefore, high *BCL2A1* expression may signify a pre-existing T-cell population poised to mount a durable attack once unleashed by ICB.

To comprehensively evaluate *BCL2A1* as a predictive bio-marker for ICB response in LUAD, a modular study design is required to move beyond simple association. We hypothesized that robust patient stratification necessitates contextualizing *BCL2A1* within a multi-dimensional framework, concurrently assessing immune checkpoint targets (CD274) and the overall inflammatory status (the HOT score) to fully capture the tumor’s susceptibility to ICB [8]. Furthermore, distinguishing bona fide immune engagement from non-specific tissue signals requires deconstructing the cellular origins of *BCL2A1*, specifically linking its expression to response-associated CD8^+^ T-cell subtypes, such as tissue-resident memory (Trm) cells, which are critical for local anti-tumor immunity [9,10,11]. Finally, to address the translational gap where genomic signatures often fail upon migrating to clinical settings, it is vital to evaluate the signature’s transferability across targeted diagnostic platforms through in silico modeling [12]. This integrative approach advances *BCL2A1* from a theoretical biological candidate to a bio-marker with potential clinical utility.

## 2. Materials and Methods

### 2.1. Study Design

This study aimed to evaluate the association of *BCL2A1* with ICB response in LUAD and to develop a composite transcriptomic model. The model integrated *BCL2A1*, *PD-L1* (CD274), and an immune activation score (HOT). These three transcriptomic features were chosen for their mechanistic complementarity and low collinearity. The HOT score is an established 27-gene immune activation signature previously reported by Foy et al. [13]. The utility of this predefined signature was confirmed in the discovery cohort (GSE161537), thereby ensuring its relevance before incorporation into our final predictive model.

To ensure reproducibility and minimize bias, analyses were structured into five modules (0, A–E). Module 0 established a preliminary characterization of *BCL2A1* through differential expression, survival, and immune-correlation analyses. Module A constructed discovery models in a bulk RNA-seq cohort using nested cross-validation. Module B validated generalizability across five independent LUAD ICB cohorts with comprehensive benchmark comparison against established immunotherapy response signatures. Module C investigated the single-cell distribution of *BCL2A1* within CD8^+^ T-cell populations, a key effector subset in ICB response, using cell–cell communication analysis to understand intercellular signaling networks. This module focused specifically on CD8^+^ T-cells and did not examine other immune cell types. Module D integrated single-cell findings into a bulk-level deconvolution framework to confirm CD8^+^ subset enrichment patterns. Module E performed in silico simulations to evaluate the clinical translatability of the Tri-Axis model across targeted transcriptomic platforms (HTG EdgeSeq, NanoString nCounter, and RT-qPCR).

This modular design ensures complete independence between analytical streams: predictive modeling development is strictly confined to bulk RNA-seq data (Modules A–B), while single-cell analysis, deconvolution, and translational simulations (Modules C–E) serve as orthogonal validation layers that provide mechanistic insights without influencing model construction or performance evaluation.

### 2.2. Study Cohorts

Bulk RNA-seq datasets analyzed in this study were obtained from the National Center for Biotechnology Information (NCBI) Gene Expression Omnibus (GEO) database (https://www.ncbi.nlm.nih.gov/geo/, accessed on 5 January 2026), including GSE161537, GSE190266, GSE190265, GSE166449, GSE126044, and GSE135222. Single-cell RNA-seq data were retrieved from GEO under accession number GSE176021.

The discovery bulk cohort consisted of 60 advanced LUAD patients treated with anti-PD-L1 therapy (GSE161537). Pre-treatment tumor biopsies underwent RNA-sequencing. External validation used five independent LUAD ICB cohorts with pre-treatment samples: GSE190266 (*n* = 56), GSE190265 (*n* = 14), GSE166449 (*n* = 22), GSE126044 (*n* = 7), and GSE135222 (*n* = 27), totaling 126 patients for comprehensive benchmark validation. All cohorts provided pre-treatment RNA-seq and radiological response annotations. The single-cell discovery dataset (GSE176021) included 11 LUAD patients treated with neoadjuvant ICB plus chemotherapy, from which CD8^+^ T cells were isolated and sequenced at single-cell resolution. Clinical response was defined according to the RECIST ver 1.1 criteria. Clinical details, including treatment regimen, stage, and driver mutation frequencies, are provided in the Supplementary Methods. Patient sex information was not consistently available across datasets and was therefore not analyzed.

#### 2.2.1. Module 0—Preliminary Bio-Marker Characterization

Differential expression analysis compared responders (R) and non-responders (NR) in the discovery cohort, adjusting for sex, tumor stage, and treatment lines. Candidate genes were prioritized by effect size and false discovery rate. Survival relevance was evaluated using Kaplan–Meier analysis and multivariate Cox regression. To test ICB specificity, *BCL2A1* was further assessed across 21 non-ICB lung adenocarcinoma cohorts through meta-analysis. Immune origin was examined by correlation with lineage markers (CD8A, PRF1, PDCD1, and FOXP3) and checkpoint molecules. Functional context was explored through gene set enrichment analysis (GSEA) [14,15,16] using Hallmark [17] pathways and immunologic gene sets from the Molecular Signatures Database (MSigDB) [18,19]. To assess immune positioning, *BCL2A1* was compared with established immune activation scores, including the FDA-endorsed Gene Expression Profile (GEP) [20], the Immunologic Constant of Rejection (ICR) [21], and the IFN-γ 6-gene signature [22,23,24]. Genes co-expressed with *BCL2A1* were subjected to functional enrichment analyses, and cellular origin was estimated using EPIC (13) deconvolution.

#### 2.2.2. Module A—Bulk Discovery Modeling

To construct predictive models, we selected three transcriptomic features: *BCL2A1*, CD274, and the HOT score, a curated 27-gene immune-hot signature [13]. Gene-level preprocessing included filtering low-abundance genes, log_2_ transformation, and robust normalization. Logistic regression with L1 regularization was implemented within a nested cross-validation framework (10 outer folds and 5 inner folds) to optimize the penalty parameters and minimize overfitting.

Model performance was evaluated using the area under the ROC curve (AUC), the area under the precision-recall curve (PR-AUC), the Brier score, and the calibration slope and intercept. The Youden index derived from out-of-fold predictions defined a locked threshold for subsequent validation. Comparative models were tested with and without *BCL2A1* and in combination with clinical covariates to assess incremental contribution. Model performance was also compared with CD274-only models, reflecting current clinical practice (see Supplementary Methods).

#### 2.2.3. Module B—External Validation Using Leave-One-Cohort-Out Design

External performance was evaluated using a leave-one-cohort-out (LOCO) framework across five independent LUAD datasets (*n* = 126). Models were fully locked after the discovery phase (Module A), including all coefficients and the Youden-derived classification threshold. No refitting or parameter adjustment was performed during external validation; locked models were applied directly to each validation cohort. Validation cohorts were selected with a harmonized therapy class and line of therapy to minimize confounding. The transcriptomic models locked in Module A were assessed using macro-validation, treating each cohort as an equally weighted validation unit to avoid size-driven bias. Model discrimination was quantified by macro-averaged AUC with cohort-level 95% CIs; secondary metrics included PR-AUC, Brier score, sensitivity, specificity, PPV, and NPV. Clinical utility was examined using decision-curve analysis across 15–50% probability thresholds and by computing Net Reclassification Improvement (NRI) and Integrated Discrimination Improvement (IDI) relative to CD274. Probability calibration was evaluated using intercept-only recalibration. Benchmark comparison incorporated four established signatures (TIDE, TIS, IPS, IFNG), each computed according to published gene definitions and macro-validated across cohorts, as detailed in the Supplementary Methods.

#### 2.2.4. Module C—Mechanistic Support from scRNA-seq and Deconvolution

To contextualize bulk findings, scRNA-seq data from 130,584 CD8^+^ T cells in GSE176021 were analyzed. These analyses were conducted at the subtype level to characterize CD8^+^ T-cell states and cellular sources of *BCL2A1* and were descriptive and hypothesis-generating rather than intended for patient-level inference of treatment response. Cells were filtered to ensure CD8^+^ purity and clustered using graph-based methods. Subtypes were annotated based on established lineage markers, yielding subsets including Trm, central memory (Tcm), proliferating exhausted (Tpex), effector memory, and terminally differentiated states. *BCL2A1* gene expression was quantified across cell subsets, and distributions were compared between major pathological responders (MPR) and non-MPR patients.

Subtypes showing consistent *BCL2A1* enrichment or enhanced communication patterns were prioritized for downstream analysis. Subtype-specific marker genes were aggregated into reference profiles, which served as the basis for bulk-level deconvolution in Module D. The statistical significance of differences in subtype composition and communication networks was evaluated using permutation testing.

Cell–cell communication analysis was performed using LIANA (v0.1.13) to investigate intercellular signaling patterns involving *BCL2A1*^+^ CD8^+^ T cells in the GSE176021 dataset. LIANA employs a rank-aggregation framework that prioritizes interactions based on multiple scoring methods (magnitude_rank, specificity_rank), reducing dependence on individual *p*-values. Two complementary analyses were conducted: (1) overall comparison of *BCL2A1*-high versus *BCL2A1*^low^ CD8^+^ T cells across 22 cell types, and (2) subset-level network analysis examining communication patterns among functional CD8^+^ T-cell subsets (proliferating, memory, effector, and exhausted populations) stratified by *BCL2A1* expression. Interaction scores (lr_means) were computed for all ligand-receptor pairs. Distribution-level statistical comparisons between *BCL2A1*^high^ and *BCL2A1*^low^ populations were performed using Mann–Whitney U tests; accordingly, reported *p*-values represent aggregate comparisons, and outputs are presented as descriptive characterization of communication patterns. Among all pathways analyzed, MIF signaling showed the highest mean interaction score and was selected for detailed network characterization (see Supplementary Methods for detailed methodology).

#### 2.2.5. Module D—Robustness and Mechanistic Support

To extend the single-cell findings to bulk RNA-seq, CD8^+^ T-cell subtype abundances in the GSE161537 discovery cohort were estimated using EPIC with custom reference matrices derived in Module C. Five matrices were used to interrogate complementary aspects of robustness: a baseline FULL reference; POS and NEG references constructed from *BCL2A1*^+^ and *BCL2A1*^−^ CD8^+^ cells to assess cell-level stratification; and two gene-ablation references (noB, dropNB) designed to remove *BCL2A1* alone or together with its correlated markers to evaluate gene-level dependency.

Robustness was examined using three approaches. First, ablation experiments tested whether subtype estimates depended on *BCL2A1*-related genes. Second, bootstrap resampling (1000 iterations) assessed stability across patient subsets. Third, Gaussian noise perturbation (±5%, 200 iterations) was used to evaluate sensitivity to technical variation. Correlations and deviation metrics between perturbed and original estimates quantified stability. This framework ensured that subtype inferences were reproducible across reference designs and resilient to sampling or noise-based perturbations. Additional methodological details are provided in the Supplementary Methods.

#### 2.2.6. Module E—Cross-Platform Feasibility Simulation

To evaluate the clinical translatability of the Tri-Axis model, we conducted in silico simulations using the LUAD ICB discovery cohort (GSE161537). Expression profiles of 29 Tri-Axis genes (*BCL2A1*, CD274, and 27 HOT-signature genes) were modeled across three representative targeted transcriptomic platforms—HTG EdgeSeq, NanoString nCounter, and RT-qPCR—according to their published technical characteristics. Platform-specific variability, dropout, and measurement bias were introduced to mimic real-world assay behavior.

Performance was assessed using correlation and agreement metrics (Spearman ρ, Cohen’s κ, Lin’s CCC, and Bland–Altman bias) with bootstrap confidence intervals. Negative-control models and stress tests examined the specificity and stability of the Tri-Axis signature under increasing technical perturbations.

This computational validation provides a realistic assessment of how the Tri-Axis framework may perform when adapted to clinical diagnostic platforms, without requiring wet-lab experimentation (see more detail in the Supplementary Methods).

### 2.3. Software and Statistical Tools

Analyses were performed using R (v4.3.3) and Python (v3.9). Data preprocessing, enrichment, and modeling were implemented with standard bioinformatics libraries. Statistical tests included log-rank and Cox regression for survival, Mann–Whitney U and Fisher’s exact for group comparisons, and DeLong tests for AUC comparisons. Package versions, parameter choices, and sensitivity checks are provided in the Supplementary Methods.

## 3. Results

### 3.1. Module 0: Preliminary Bio-Marker Characterization

#### 3.1.1. *BCL2A1* Emerges from Prognostic Filtering of Differentially Expressed Genes

**Figure 1:**
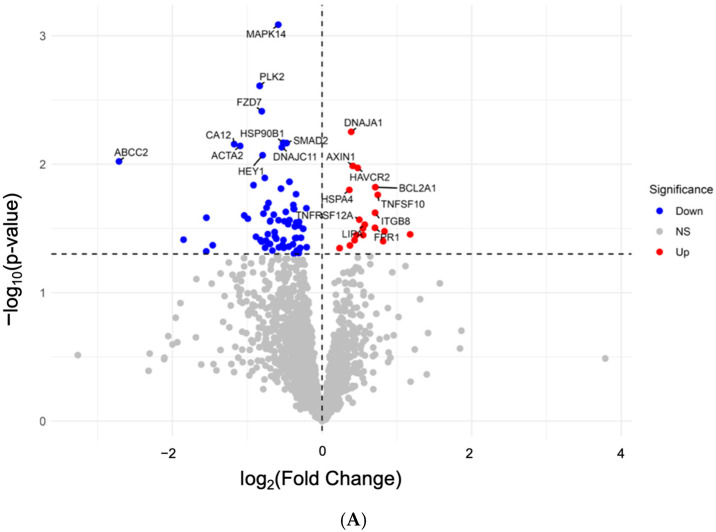

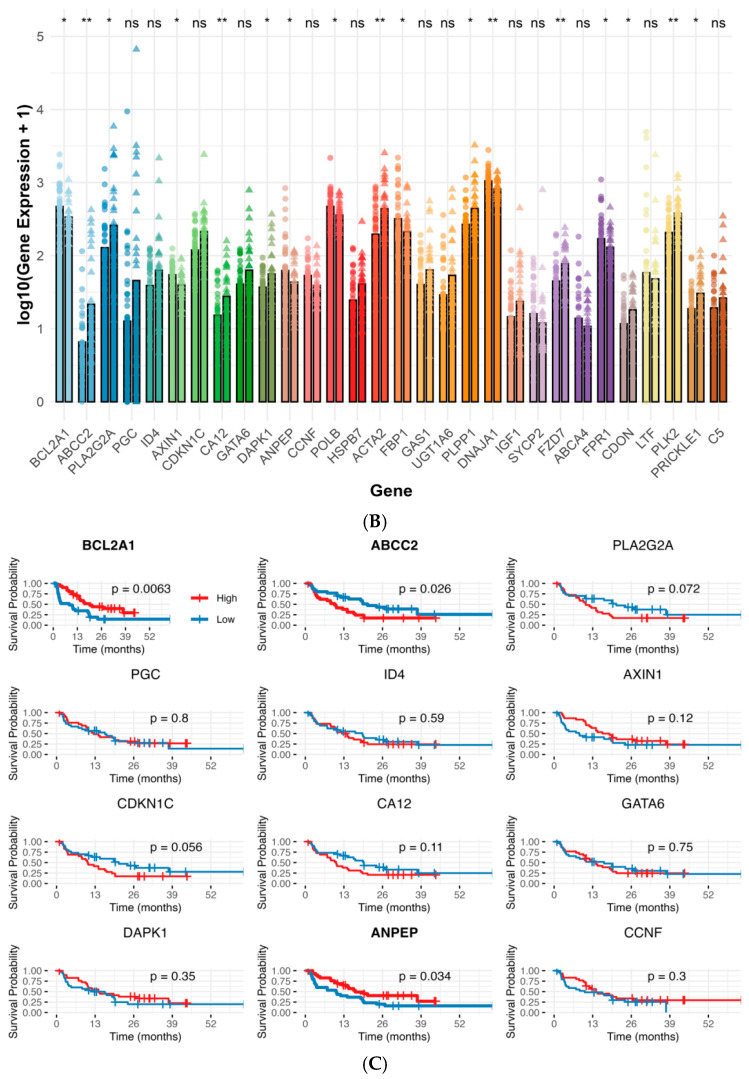
Differential expression and prognostic relevance of *BCL2A1* and related genes in the ICB-treated LUAD discovery cohort. (**A**) Volcano plot displaying transcriptome-wide differential expression analysis. Each dot represents one gene, with red indicating genes upregulated in responders (Up), blue indicating genes downregulated in responders (Down), and gray indicating non-significant changes (NS). Selected genes with significant fold change and adjusted *p*-values are labeled. (**B**) Bar plots showing normalized expression (log_10_[gene expression +1]) of selected candidate genes, stratified by responder (circle) versus non-responder (triangle) groups. Bars denote mean ± SEM. Statistical significance was assessed by Mann–Whitney U test, with multiple testing correction (BH-FDR). ns, not significant; * *p* < 0.05; ** *p* < 0.01. (**C**) Kaplan–Meier survival curves for the top 12 differentially expressed genes (DEGs) ranked by log fold change between responders and non-responders in GSE161537. Patients were stratified into high- and low-expression groups using median cutoffs. *p*-values were computed using the log-rank test.

In the ICB-treated discovery cohort (*n* = 60), differential expression analysis between responders and non-responders identified 30 genes with *p* < 0.05 (Figure 1A,B), of which 12 genes achieving FDR < 0.05 underwent further survival analysis. Among these candidates, *BCL2A1* was significantly associated with improved overall survival (HR = 0.43, *p* < 0.05; Figure 1C). Notably, meta-analysis across 21 non-ICB adenocarcinoma cohorts demonstrated an opposing prognostic trend (HR = 1.07, 95% CI: 1.01–1.14, *p* = 0.0203; I^2^ = 0%; Figure S1) [25,26,27,28,29,30,31,32,33,34,35,36,37,38,39,40,41,42,43,44,45], suggesting that the survival benefit of *BCL2A1* is context-specific to immunotherapy settings. *BCL2A1* expression showed no significant association with sex (*p* = 0.72), tumor stage (*p* = 0.45), or line of therapy (*p* = 0.39) (Table S1).

#### 3.1.2. Immune Correlation and Pathway Enrichment

Correlation analysis revealed strong positive associations between *BCL2A1* and immune effector genes: PRF1 (r = 0.62), GZMB (r = 0.56), CD8A (r = 0.60), PDCD1 (r = 0.69), and key regulators including HAVCR2, LAG3, TBX21, and IFNG (r > 0.48; all *p* < 0.001). In contrast, CD274 showed a weak, non-significant correlation (r = 0.12, *p* = 0.35) (Table S2). Stratified analysis demonstrated that responders retained strong co-expression patterns (e.g., PRF1 r = 0.60, PDCD1 r = 0.55), unlike non-responders (Table S3), suggesting a *BCL2A1*-linked immune activation program specific to therapeutic response.

**Figure 2:**
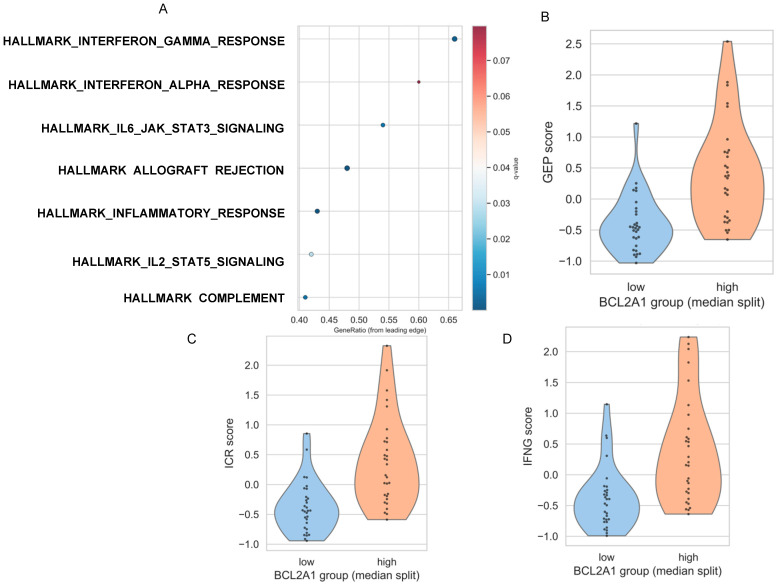
Immunological associations of *BCL2A1* expression in bulk LUAD tumors. (**A**) Enrichment of immune-related Hallmark gene sets was positively correlated with *BCL2A1* expression, as assessed by GSEA using Spearman’s rank correlation. Dot size indicates normalized enrichment score (NES); color represents FDR-adjusted *p*-value. (**B**–**D**) Violin plots showing the distribution of immune activation scores stratified by *BCL2A1* expression (median split): (**B**) 18-gene T-cell inflamed signature (GEP), (**C**) Immunologic Constant of Rejection (ICR), and (**D**) IFNG expression score. Each point represents one tumor sample.

GSEA revealed that *BCL2A1*-high patients were enriched for immune-related Hallmark pathways: Allograft Rejection (NES = 1.77, q = 8.5 × 10^−9^), Inflammatory Response (NES = 1.59, q = 5.9 × 10^−5^), Interferon Gamma Response (NES = 1.49, q = 0.0017), IL6-JAK-STAT3 (NES = 1.44, q = 0.0053), and Complement (NES = 1.40, q = 0.0051) (Figure 2A). *BCL2A1* correlated significantly with established hot tumor signatures, including T-cell inflamed GEP (r = 0.56), ICR (r = 0.59), and the IFN-γ 6-gene signature (r = 0.49) (all *p* < 0.001), with expression significantly elevated in GEP/ICR/IFN-γ-high tumors (*p* < 0.001; Figure 2B–D).

#### 3.1.3. Co-Expression Network and Cellular Localization

**Figure 3:**
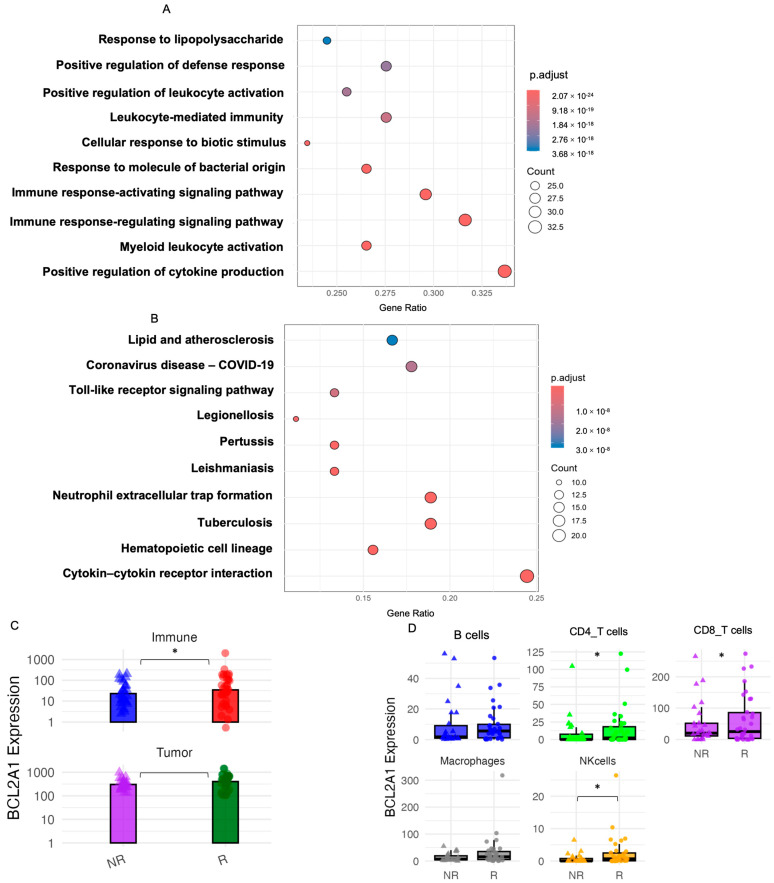
Functional enrichment and immune compartmentalization of *BCL2A1*-associated signals. Gene set enrichment analysis (GSEA) of the top 100 genes positively co-expressed with *BCL2A1* in the discovery cohort (GSE161537). (**A**) GO Biological Process (GO-BP) terms enriched for immune activation, cytokine signaling, and myeloid lineage responses. (**B**) KEGG pathways implicating lipid metabolism, inflammation, and cytokine receptor signaling. Bubble size represents gene ratio; color scale reflects adjusted *p*-values. EPIC-based deconvolution of *BCL2A1* expression across ICB response groups. (**C**) Compartment-level estimates of *BCL2A1* expression in immune and tumor components, stratified by response (NR = non-responder; R = responder), * *p* < 0.05, Blue and red denote non-responders (NR) and responders (R) in the immune compartment, respectively, while purple and green denote non-responders and responders in the tumor compartment. (**D**) Subtype-level *BCL2A1* expression in major immune lineages (B cells, CD4^+^ T cells, CD8^+^ T cells, macrophages, and NK cells), estimated separately for NR and R groups, * *p* < 0.05.

Among the top 100 co-expressed genes, GO enrichment analysis highlighted cytokine production (GO:0001819, q = 1.21 × 10^−21^), myeloid leukocyte activation (GO:0002274, q = 1.85 × 10^−21^), and immune signaling regulation (GO:0002764, q = 1.58 × 10^−19^) (Figure 3A). KEGG pathway analysis identified cytokine–cytokine receptor interaction (hsa04060, q = 3.82 × 10^−12^), hematopoietic cell lineage (hsa04640, q = 2.29 × 10^−11^), and tuberculosis pathway (hsa05152, q = 3.39 × 10^−11^) among the top-enriched pathways (Figure 3B). EPIC deconvolution demonstrated that *BCL2A1* was upregulated in immune compartments of responders; tumor-compartment expression showed no statistically significant association with response (Figure 3C). Among immune subsets, NK cells exhibited the highest expression, followed by CD8^+^ and CD4^+^ T cells, with modest but significant differences between responders and non-responders (Figure 3D).

### 3.2. Module A: Bulk Discovery Modeling

#### 3.2.1. Performance of Transcriptomic Models

**Figure 4:**
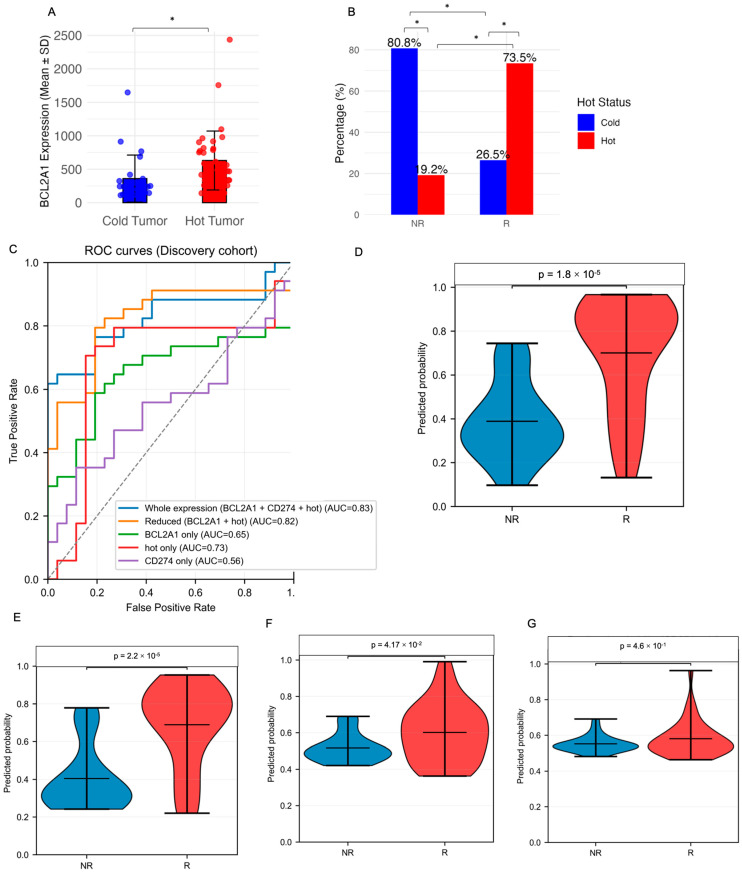
*BCL2A1* expression associates with a 27-gene immune-hot microenvironment and ICB response prediction models. (**A**) *BCL2A1* expression levels in tumors stratified by immune-hot vs. immune-cold status, as defined by HOT score (two-sided Wilcoxon test), * *p* < 0.05. (**B**) Distribution of HOT status across responders (R) and non-responders (NR), indicating a higher prevalence of immune-hot tumors in responders (Fisher’s exact test), * *p* < 0.05. (**C**) ROC curves of logistic regression models predicting ICB response in the discovery cohort. The full model (*BCL2A1* + CD274 + HOT score) outperformed reduced variants. Predicted probabilities from top models, stratified by ICB response group. (**D**) Full model using all three variables (AUC = 0.83). (**E**) Reduced model (AUC = 0.82). (**F**) *BCL2A1* only (AUC = 0.75). (**G**) CD274 only (AUC = 0.56). All models show significantly higher predicted probability in responders (two-sided Wilcoxon test). In all panels, colors indicate biological grouping: blue denotes cold tumors or non-responders (NR), and red denotes hot tumors or responders (R), as appropriate.

*BCL2A1* expression was significantly elevated in immune-hot tumors as defined by a 27-gene HOT score (Figure 4A), which were enriched among responders (Figure 4B). Among tested models, the tri-marker combination (*BCL2A1* + CD274 + HOT) achieved optimal performance: out-of-fold ROC-AUC = 0.826 (95% CI: 0.704–0.925), PR-AUC = 0.900, and Brier score = 0.166 (Figure 4C,D; Table S4). The reduced model (*BCL2A1* + HOT) performed comparably (AUC = 0.822, PR-AUC = 0.886), while removal of *BCL2A1* resulted in marked performance decrements: HOT + CD274 (AUC = 0.756), HOT alone (AUC = 0.727), and CD274 alone (AUC = 0.557) (Figure 4E–G).

#### 3.2.2. Immune-Partitioned Expression and Clinical Augmentation

To dissect cellular origin, models were tested using immune-partitioned expression derived from EPIC deconvolution. These performed poorly: *BCL2A1*_Immune (AUC = 0.462), CD274_Immune (AUC = 0.447), and even their combination with HOT (AUC = 0.735) underperformed compared to the whole-tissue model (AUC = 0.826; *p* = 0.085, DeLong test) (Table S5), suggesting that predictive signals are better retained in bulk expression. The addition of binary clinical covariates (line, stage) provided no improvement; line_bin (AUC = 0.419), stage_bin (AUC = 0.449), and the augmented model (AUC = 0.822) failed to outperform transcriptomic models alone (Tables S6 and S7), with net reclassification index changes near zero or negative (e.g., –0.029) (Table S8). Pairwise statistical tests confirmed that the whole-tissue model significantly outperformed single-marker, immune-partitioned, and immune-fraction alternatives (*p* < 0.05), while the reduced model remained statistically comparable (*p* = 0.886), reinforcing *BCL2A1*’s additive value over CD274.

### 3.3. Module B: External Validation

**Figure 5:**
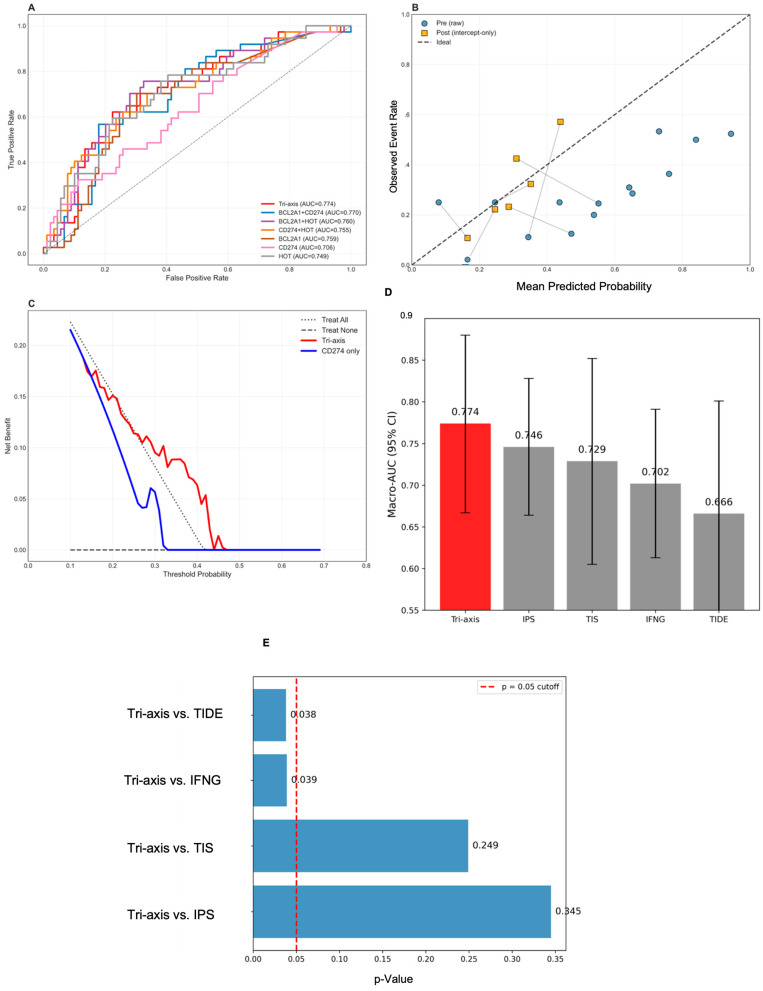
Cross-cohort validation and benchmarking of the Tri-Axis model. (**A**) Macro ROC curve for leave-one-cohort-out (LOCO) validation across five external cohorts, showing an overall AUC of 0.774. (**B**) Calibration plots before and after intercept-only recalibration. The raw model underestimated probability in high-risk regions, which was corrected by intercept adjustment. (**C**) Decision-curve analysis demonstrating higher net clinical benefit for the Tri-Axis model across a broad range of threshold probabilities relative to CD274 alone. (**D**) Comparison of macro-AUC across benchmark signatures, confirming that the Tri-Axis model achieved the highest pooled discrimination (Macro-AUC = 0.774). The tri-axis model is highlighted in red, while benchmark models (IPS, TIS, IFNG, and TIDE) are shown in gray. (**E**) Statistical comparison of the Tri-Axis model versus reference signatures using the DeLong test; the dashed line indicates the *p* = 0.05 significance cutoff.

To assess generalizability, locked transcriptomic models were evaluated across independent LUAD datasets using a leave-one-cohort-out (LOCO) framework. The Tri-Axis bio-marker (*BCL2A1* + CD274 + HOT) achieved strong external performance with a macro-AUC of 0.774 (95% CI: 0.650–0.898; Figure 5A), significantly outperforming CD274 alone (0.706, *p* = 0.036). Performance remained consistent despite substantial cohort heterogeneity (I^2^ = 84%). Secondary metrics were favorable: PR-AUC = 0.595, Brier score = 0.246, sensitivity = 0.859, specificity = 0.513, and NPV = 0.905 (Table S9). Intercept-only recalibration reduced systematic overestimation (intercept: −1.138→−0.137; Brier: 0.219→0.162) (Figure 5B; Table S10). Decision-curve analysis demonstrated higher net benefit than CD274 across all probability thresholds, particularly at 15–20% (Figure 5C; Table S11). Clinical reclassification improved markedly (NRI = 0.218; IDI = 0.185), corresponding to correctly reclassifying approximately one in five patients (Table S12).

In benchmark comparisons against established signatures, the Tri-Axis bio-marker delivered the highest macro-AUC (0.774), outperforming IPS (0.746), TIS (0.729), IFNG (0.702), and TIDE (0.666) (Figure 5D). Pairwise cohort-level testing confirmed significant gains over TIDE and IFNG (*p* < 0.04) (Figure 5E). Clinical utility analyses further supported improved discrimination (IDI = 0.190; NRI = 0.108 at 15% threshold) and higher net benefit at 25% threshold (Figure S2).

### 3.4. Module C: Single-Cell Characterization

#### 3.4.1. CD8^+^ T-Cell Clustering and *BCL2A1* Distribution

**Figure 6:**
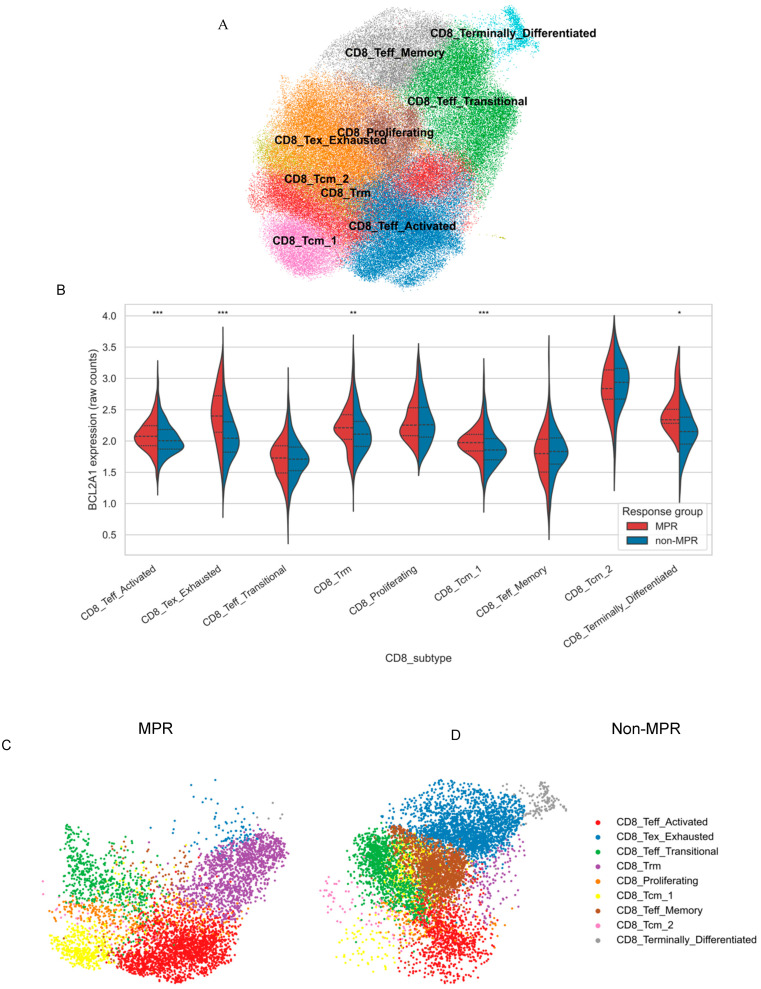
*BCL2A1* expression is enriched in specific CD8^+^ T-cell states associated with pathological response (Module C). (**A**) UMAP projection of CD8^+^ T cells from the GSE176021 cohort, annotated into nine functional subtypes, including TRM, Tcm, Teff (activated, memory, and transitional), proliferating, exhausted (Tex), and terminally differentiated states. (**B**) Violin plots showing *BCL2A1* expression (log_10_ TPM) across subtypes, stratified by pathological response group, * *p* < 0.05; ** *p* < 0.01; *** *p* < 0.001. Dashed lines within the violin plots indicate the median and interquartile range (25th–75th percentiles). UMAP projections of CD8^+^ cells from MPR (**C**) and non-MPR (**D**) patients separately, highlighting compositional differences in subtype distribution (FDR < 0.05 for CD8_Tcm_1, CD8_Teff_Activated, CD8_Trm, CD8_Tex_Exhausted, CD8_Terminally_Differentiated).

Unsupervised analysis of 130,584 CD8^+^ T cells from GSE176021 identified transcriptionally distinct clusters annotated into functional phenotypes, including activated effector, Trm, proliferating, Tcm, exhausted, memory-like, and terminally differentiated states (Figure 6A). Stratification of *BCL2A1*-expressing cells by pathological response revealed distinct subtype distributions: Trm and proliferating subsets were preferentially enriched in MPR patients, suggesting association with effective immunotherapeutic activity (Figure 6B). Mean *BCL2A1* expression was highest within Trm and proliferating clusters, whereas exhausted and memory-like subtypes exhibited minimal expression (Figure 6C,D), indicating functional selectivity.

For downstream deconvolution, subtype-specific gene signatures were derived from seven CD8^+^ subtypes showing either significant differential abundance in MPR versus non-MPR within the *BCL2A1*^+^ compartment or differential *BCL2A1* expression (adjusted *p* < 0.01). High-confidence markers were filtered by log_2_FC > 1, AUROC > 0.7, and adjusted *p* < 0.01, yielding 270 unique genes after merging and deduplication. Permutation-based validation (1000× iterations) confirmed that Trm and proliferating subsets showed observed differences exceeding the null distribution 95% confidence intervals, indicating statistically non-random enrichment in MPR patients (Table S13).

#### 3.4.2. Cell–Cell Communication Network Analysis

Cell–cell communication analysis using the LIANA methodology identified 1222 interactions involving *BCL2A1*^high^ CD8^+^ T cells, compared with 1175 for *BCL2A1*^low^ counterparts. When acting as signal sources, *BCL2A1*^high^ cells demonstrated significantly stronger interaction scores (mean = 2.91 vs. 2.79; Δ = 0.12, Mann–Whitney *p* = 0.0278; Figure S3A), indicating enhanced capacity for outgoing intercellular signaling. Network analysis revealed *BCL2A1*^high^ proliferating T cells as central communication hubs, with the highest connectivity to memory, terminally differentiated, and effector subsets (Figure S3B). *BCL2A1*^high^ subsets also demonstrated enhanced connectivity to B cells and plasma cells (Figure S3C), while erythrophagocytic macrophages emerged as central coordinators in CD8-myeloid networks (Figure S3D). MIF signaling exhibited the highest mean interaction scores when *BCL2A1*^high^ cells served as either sources (mean = 6.08) or targets (mean = 5.91), with MIF-myeloid crosstalk achieving peak intensity (mean = 6.53), representing 2–3-fold increases compared to other pathways, including HLA-E, TNF, and FAM3C (Figure S4).

### 3.5. Module D: Bulk Deconvolution and Robustness

**Figure 7:**
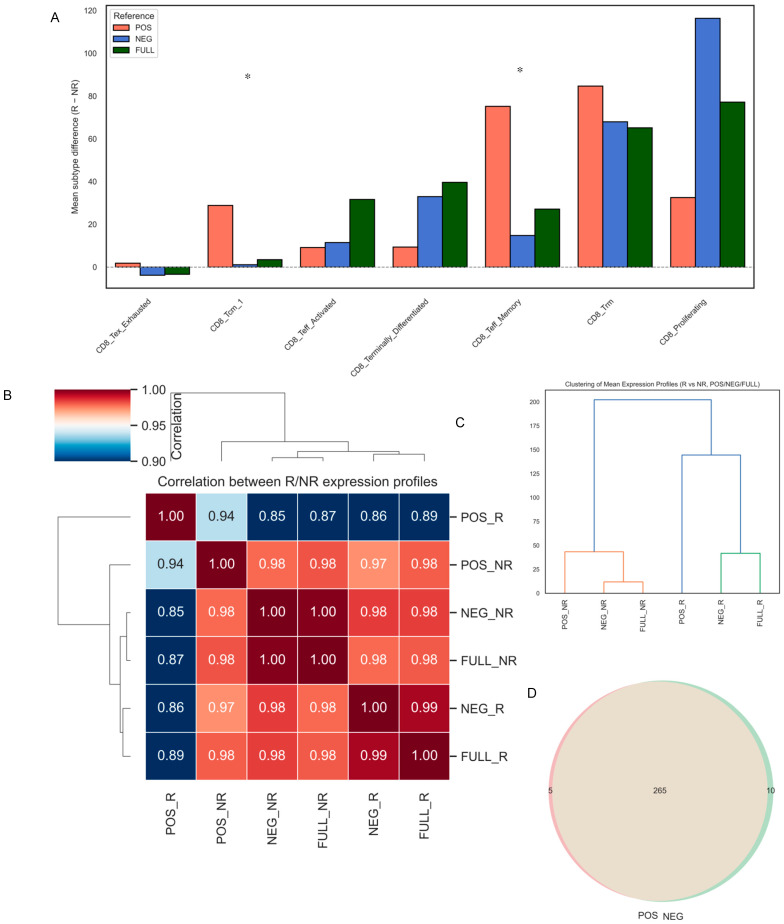
Robustness and internal concordance of *BCL2A1*-stratified CD8^+^ reference matrices (Module D). (**A**) Bar plot showing differences in subtype fractions between responders (R) and non-responders (NR) across three reference matrices (POS, NEG, FULL), * *p* < 0.05. (**B**) Heatmap of pairwise Spearman correlation coefficients between mean expression profiles of R/NR groups across all reference matrices. (**C**) Hierarchical clustering of reference-wise expression profiles supports distinct grouping of responder-derived profiles (POS_R, FULL_R, NEG_R) versus non-responder-derived profiles. (**D**) Venn diagram showing gene content overlap between the POS and NEG reference matrices.

EPIC deconvolution was performed on GSE161537 using five reference matrices (FULL, POS, NEG, noB, dropNB) designed to test biological and technical robustness. Using the FULL matrix, responders showed significant enrichment of Teff_Memory (Δ = 75.2, MW_padj = 0.020) and Tcm_1 (Δ = 28.8, MW_padj = 0.020), with consistent but non-significant increases in Trm and proliferating subsets (Figure 7A; Table S14). Re-analysis with POS and NEG matrices produced highly concordant results (Pearson r ≥ 0.85) and preserved responder-associated trends (Figure 7B). Given that *BCL2A1*^+^ cells constitute approximately 10% of the CD8^+^ population, similarities between POS and NEG matrices indicate that *BCL2A1* marks an activation spectrum rather than a distinct lineage.

Gene-level robustness was confirmed using noB and dropNB matrices. Removing *BCL2A1* alone (noB) produced subtype profiles nearly identical to FULL (Spearman ρ > 0.90; Table S15). The more stringent dropNB condition yielded moderate reductions (ρ ≈ 0.4–0.6) but retained responder–non-responder patterns. Predictive accuracy remained unaffected (FULL AUC = 0.688; noB = 0.714; dropNB = 0.721; all DeLong *p* > 0.95) (Table S16). Signature overlap between the POS and NEG matrices revealed 265 shared genes, with only 5 POS-specific and 10 NEG-specific markers (Figure 7C,D; Table S17). Bootstrap resampling (1000 iterations) produced narrow interquartile ranges (IQR ≤ 0.038) and low standard deviations (SD ≤ 0.016), while Gaussian noise injection (±5%, 200 iterations) resulted in minimal absolute shifts (mean ≤ 0.025) across all subtypes (Table S18).

### 3.6. Module E: Cross-Platform Feasibility Simulation

**Figure 8:**
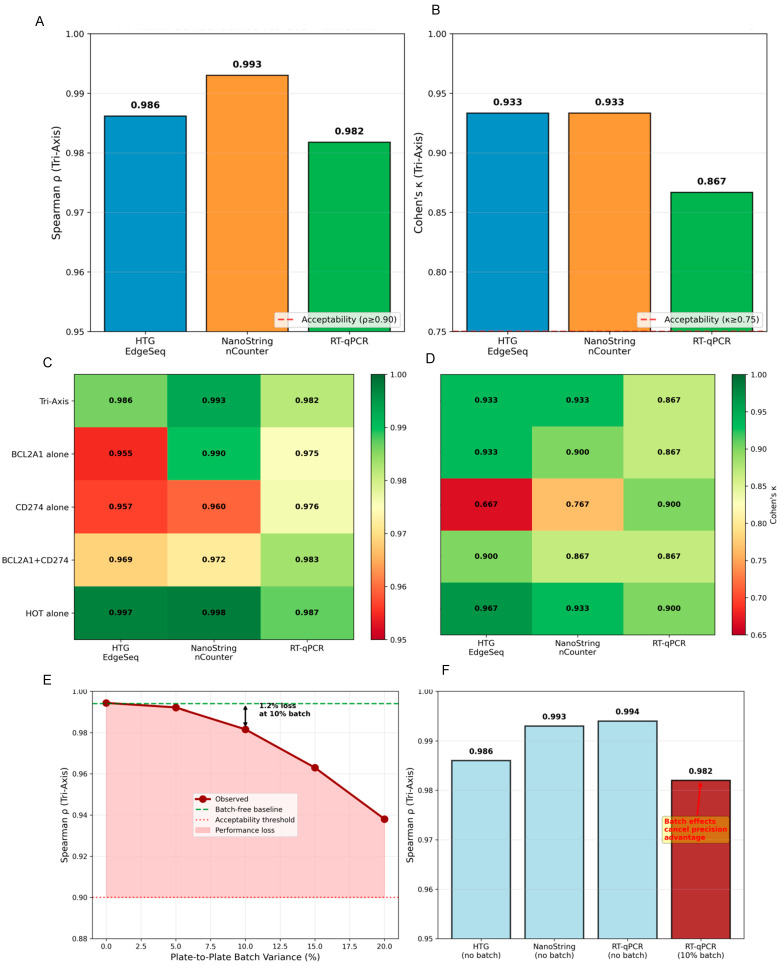
Cross-platform concordance and RT-qPCR batch effect. (**A**) Schematic comparison of platform-specific concordance measured by Spearman ρ across the three simulated transcriptomic platforms (HTG EdgeSeq, NanoString nCounter, and RT-qPCR). (**B**) Cohen’s κ agreement across the same platforms, with horizontal dashed lines indicating pre-defined acceptance thresholds. (**C**) Heatmap illustrating cross-platform rank correlation for the Tri-Axis and negative-control signatures (five total) under identical simulation settings. (**D**) Heatmap showing corresponding Cohen’s κ values for the same set of signatures, highlighting relative robustness and specificity. (**E**) Stress-test simulation assessing the impact of increasing RT-qPCR batch variance (0–20%) on correlation performance; the shaded region represents the acceptable tolerance range. (**F**) Comparison of simulated batch-free versus batched RT-qPCR performance relative to digital-counting platforms.

All clinical platforms achieved acceptable concordance with the RNA-seq reference (Table S19; Figure 8A,B): NanoString (ρ = 0.993, 95% CI: 0.976–0.997; κ = 0.933), HTG (ρ = 0.986, CI: 0.976–0.997; κ = 0.933), and RT-qPCR (ρ = 0.982, CI: 0.960–0.989; κ = 0.867). Single-gene signatures showed degraded performance (Figure 8C,D; Table S20): *BCL2A1* demonstrated platform dependency (NanoString ρ = 0.990; HTG ρ = 0.955), CD274 showed precision dependency (RT-qPCR ρ = 0.976 vs. digital platforms ρ ≈ 0.96), while HOT remained universally robust (ρ = 0.987–0.998), confirming multi-gene averaging benefits.

Batch effects dominated technical variance: RT-qPCR batch-free achieved ρ = 0.994 (best overall), declining to ρ = 0.982 with 10% batch variance and ρ = 0.938 with 20% variance (Figure 8E; Table S21). Amplification efficiency variance (±20%) had a negligible impact (Δρ < 0.002), demonstrating that systematic bias outweighs random noise (Figure 8F). Digital platforms maintained ρ > 0.95 at 30% CV and ρ > 0.97 at 15% dropout. HOT 50% gene ablation maintained ρ ≥ 0.979, confirming signature redundancy (Table S22). All platforms showed negligible systematic bias (<0.002 z-score) with Lin’s CCC > 0.98 (NanoString: 0.989) and stable threshold agreement across ±10% range (κ variation < 0.05 for HTG/NanoString, < 0.07 for RT-qPCR) (Table S22).

## 4. Discussion

This study investigated *BCL2A1* as an associative bio-marker of ICB response in LUAD using a modular analytical framework that integrates bulk and single-cell transcriptomic data. Our findings suggest *BCL2A1* may be associated with favorable ICB outcomes, particularly when combined with *PD-L1* (CD274) and a pre-defined immune activation signature (HOT score). The tri-marker model demonstrated reproducible associations across multiple independent datasets, with mechanistic insights from CD8^+^ T-cell profiling providing a plausible biological context [46].

In the discovery cohort, *BCL2A1* expression was elevated in ICB responders, and functional enrichment revealed immune pathway activation consistent with prior reports of *BCL2A1*’s anti-apoptotic function in T-cell persistence [47,48,49,50]. Notably, a meta-analysis of 21 non-ICB LUAD cohorts revealed a modest association between high *BCL2A1* and poorer prognosis, likely reflecting tumor-intrinsic anti-apoptotic programs. In contrast, under ICB therapy, elevated *BCL2A1* in CD8^+^ T cells—particularly within Trm and Tpex subsets—appeared associated with improved outcomes, underscoring *BCL2A1*’s context-dependent roles and the importance of compartment-specific interpretation [51].

The tri-marker model incorporating *BCL2A1*, *CD274*, and the HOT score demonstrated a stronger association with ICB response than CD274 alone [52,53]. While PD-L1 primarily reflects adaptive immune resistance at the tumor–immune interface [54,55,56], *BCL2A1* may serve as a surrogate marker of CD8^+^ T-cell survival capacity. The HOT score provides a third orthogonal dimension that captures the broader inflammatory context.

External validation across five independent LUAD cohorts (n = 126) using the leave-one-cohort-out (LOCO) framework suggested the model may generalize beyond discovery settings, achieving a macro-AUC of 0.774 (95% CI: 0.650–0.898), which compared favorably to CD274 alone (0.706) and established signatures including TIDE (0.666), IFNG (0.702), TIS (0.729), and IPS (0.746) [3]. Reclassification metrics (NRI = 0.218; IDI = 0.185) indicated potential for improved patient stratification. However, findings require cautious interpretation given cohort heterogeneity (I^2^ = 84%) and relatively small validation sample sizes [57].

scRNA-seq analysis provided a biological context for the bulk-level associations. The single-cell dataset (GSE176021) was derived from a neoadjuvant chemo-immunotherapy setting with major pathological response (MPR) as the endpoint, which differs from the advanced/metastatic setting of bulk RNA-seq validation cohorts evaluated by RECIST. While both endpoints reflect effective immune-mediated tumor control, direct extrapolation of single-cell findings to metastatic disease should be interpreted cautiously; these findings are presented as mechanistic support rather than independent clinical validation [51,58]. Additionally, both neoadjuvant and metastatic cohorts include stage III patients, supporting biological comparability. The use of post-treatment samples—collected after two cycles of neoadjuvant ICB plus chemotherapy—enabled the capture of early treatment-induced CD8^+^ T-cell dynamics while preserving baseline characteristics that had not yet been fully remodeled. *BCL2A1* expression appeared enriched in memory-like, proliferative, and tissue-resident CD8^+^ subsets, including Tpex and Trm cells implicated in ICB responsiveness [59], offering a potential biological rationale for the observed signals.

Cell–cell communication analysis provided mechanistic support. *BCL2A1*^high^ CD8^+^ T cells displayed significantly higher outgoing signaling (*p* = 0.0278), with the MIF pathway emerging as dominant (mean = 6.08 as source; 6.53 in CD8–myeloid crosstalk). *BCL2A1*^high^ proliferating cells functioned as coordination hubs, linking memory, effector, and terminally differentiated states, and showed strengthened interactions with plasma cells, which were associated with favorable outcomes [60,61,62,63,64,65,66,67]. The MIF-CD74 axis exhibited interaction strengths 2–3× higher than other pathways, consistent with shared anti-apoptotic programs [68,69]. These results suggest bulk *BCL2A1* reflects coordinated immune activation, though functional studies are required to establish causality.

Co-expression patterns suggest *BCL2A1* may participate in NF-κB and cytokine signaling pathways. Prior studies demonstrated that NF-κB can upregulate *BCL2A1* in activated T cells and protect CD8^+^ T cells from apoptosis [70,71,72], consistent with *BCL2A1* potentially functioning as a survival factor in immune-active subsets [73,74,75,76,77]. However, without functional validation, this interpretation remains speculative.

EPIC deconvolution with customized CD8^+^ reference panels [7,78] demonstrated *BCL2A1*-associated subtypes showing significant responder enrichment after FDR correction. Ablation experiments confirmed subtype estimates remained stable after removing *BCL2A1* alone (noB) or with correlated markers (dropNB), indicating enrichment signals do not depend circularly on *BCL2A1*. Bootstrap resampling and noise perturbation confirmed the stability of the estimate. Preliminary melanoma analyses [79] showed modest performance (AUC ≈ 0.70), but pan-cancer extrapolation remains speculative.

In silico platform simulations revealed that NanoString nCounter demonstrated the highest concordance (ρ = 0.993) with negligible bias, suggesting suitability for clinical adaptation. HTG EdgeSeq showed excellent agreement (ρ = 0.986) as a cost-efficient alternative. RT-qPCR, despite its high precision, exhibited substantial batch-effect sensitivity. The “RT-qPCR paradox”—where 10% batch variance reduced correlation by 1.2% while ±20% amplification deviations affected it by <0.2%—challenges conventional assay hierarchies, suggesting that minimizing systematic bias may be more critical than reducing random noise [80,81,82,83].

Marker-level analyses highlighted gene-specific platform dependencies: *BCL2A1* appeared dropout-sensitive, CD274 showed precision-dependency, while HOT maintained uniformly high stability (ρ ≥ 0.987), reflecting the benefits of multi-gene averaging. NanoString emerges as potentially optimal for clinical validation and regulatory translation [84,85].

This study has significant limitations. External validation relied on five cohorts of varying sizes (*n* = 7–56), constraining precision and generalizability. Single-cell findings derived from a neoadjuvant cohort may differ from metastatic settings. Batch effects may persist despite ComBat correction. Most discovery patients received nivolumab monotherapy with rare driver mutations, potentially limiting applicability. This study focused on CD8^+^ T cells; the contribution of other *BCL2A1*-expressing populations, such as NK cells, warrants investigation in future studies. Sex-based analysis and clinical confounders, including TMB, smoking status, EGFR/ALK alterations, and protein-level PD-L1 IHC, were not consistently available and could not be systematically adjusted for; future prospective studies incorporating these variables are warranted. All analyses are associative; functional validation remains essential. The in silico platform simulations, though based on published specifications, simplify real-world factors and should be viewed as theoretical benchmarks pending wet-lab validation.

Future work should prioritize pilot studies (100–200 samples) to establish clinically meaningful *BCL2A1* cutoffs in accordance with REMARK guidelines [86]. Validation should proceed along technical (RNA-seq versus NanoString/HTG) and clinical (prospective cohort n ≥ 100) tracks. Functional studies—such as CRISPR modulation or adoptive transfer experiments—will establish whether *BCL2A1* plays a causal role. Multi-omic integration with proteomics or spatial transcriptomics could refine predictive utility [87,88].

## 5. Conclusions

*BCL2A1* expression in CD8^+^ T cells aligns with favorable immune contexture in LUAD and is associated with improved ICB outcomes. Single-cell profiling showed *BCL2A1* enrichment in tissue-resident memory and proliferating subsets that expand in responders and engage in coordinated cell–cell communication. Combined with *CD274* and the HOT score, *BCL2A1* forms a tri-marker model that performs consistently across cohorts. Although limited by sample size, heterogeneity, and associative nature, convergent evidence supports further investigation. Prospective validation and functional studies will clarify the clinical potential of this bio-marker framework.

## Data Availability

All datasets analyzed in this study are publicly accessible through the National Center for Biotechnology Information (NCBI) Gene Expression Omnibus (GEO) database (https://www.ncbi.nlm.nih.gov/geo/, accessed on 5 January 2026). Bulk RNA-seq cohorts included GSE161537, GSE190266, GSE190265, GSE166449, GSE126044, and GSE135222. Single-cell RNA-seq data were obtained from GSE176021. Accession numbers are provided in the Materials and Methods and Supplementary Materials. Further inquiries can be directed to the corresponding author.

## Supplementary Materials

The following supporting information can be downloaded at https://www.mdpi.com/article/10.3390/diagnostics16030475/s1, Figure S1. Meta-analysis of BCL2A1 expression and prognosis in lung adenocarcinoma (LUAD). Figure S2. Comparative evaluation of the Tri-axis biomarker against established transcriptomic signatures. Figure S3. CD8^+^ T-cell communication networks and key ligand–receptor interactions. Figure S4. MIF signaling network heatmap. Table S1. Multivariable logistic regression of BCL2A1 expression with ICB response and clinical covariates. Table S2. Correlation between BCL2A1 expression and immune-checkpoint or effector markers in the discovery LUAD cohort. Table S3. Correlation of BCL2A1 with immune and checkpoint markers stratified by response group. Table S4. Out-of-fold performance metrics for baseline predictive models in the GSE161537 cohort. Table S5. Pairwise DeLong test comparing AUCs between baseline models and the full model. Table S6. Out-of-fold AUC performance for augmented models including clinical covariates. Table S7. DeLong test for pairwise AUC comparisons among augmented models. Table S8. Net reclassification improvement (NRI) for augmented models compared to baseline models. Table S9. Macro discrimination metrics across LOCO multicohort external validation. Table S10. Calibration and Brier score metrics before and after intercept-only recalibration. Table S11. Net benefit from decision curve analysis after intercept-only recalibration. Table S12. Net reclassification improvement (NRI) and IDI for benchmark models compared with CD274 baseline. Table S13. Permutation test results comparing CD8^+^ T-cell subtype frequencies between MPR and non-MPR groups. Table S14. Summary of CD8^+^ subtype enrichment in responders vs non-responders across POS, NEG, and FULL reference matrices. Table S15. Robustness of CD8^+^ subtype proportions to marker ablation. Table S16. Predictive performance of logistic regression models using ablated CD8^+^ subtype signatures. Table S17. Exclusive and shared top-ranking genes from POS and NEG CD8^+^ reference signatures. Table S18. Stability of CD8^+^ subtype estimates under bootstrap and noise perturbation. Table S19. Cross-platform concordance of the Tri-axis signature. Table S20. Negative-control signature performance across platforms. Table S21. Stress-test simulation of platform robustness. Table S22. Agreement analysis and response-stratified performance.

## Abbreviations

The following abbreviations are used in this manuscript:

BCL2A1	B-cell lymphoma 2-related protein A1
EPIC	Estimating the proportions of immune and cancer cells
ICB	Immune checkpoint blockade
IDI	Integrated Discrimination Improvement
LOCO	Leave-one-cohort-out
LUAD	Lung adenocarcinoma
MPR	Major pathological response
NR	Non-responder
NRI	Net reclassification improvement
NSCLC	Non-small cell lung cancer
PD-1/PD-L1	Programmed cell death protein 1/Programmed death-ligand 1
R	Responder
scRNA-seq	Single-cell RNA sequencing
