# Supplementary material for: BCL2A1high CD8+ T Cells Are a Survival-Associated Predictor of Immune Checkpoint Blockade Response in Lung Adenocarcinoma
Source: Diagnostics (Basel). 2026 Feb 3;16(3):475. doi: 10.3390/diagnostics16030475 (PMC12896543; doi:10.3390/diagnostics16030475)
Supplement: Supplementary file 1 [file diagnostics-16-00475-s001.zip › diagnostics-4108684-supplementary.pdf]

Supplementary Methods

Study Cohorts (Extended)

The discovery cohort GSE161537 included 82 patients with non-small-cell lung cancer, of whom 60 cases with lung adenocarcinoma were retained for analysis. The median age was 63 years, with 58% being male. Most patients received nivolumab (94%), and a minority received pembrolizumab (6%) as first- or second-line therapy. The prevalence of actionable driver alterations such as EGFR, MET, or ROS1 was below 5%, and disease stages ranged from IB to IV.

The neoadjuvant cohort GSE176021 comprised 11 patients with lung adenocarcinoma who underwent tumor sampling after two cycles of nivolumab combined with chemotherapy. All cases were stage IIIB–IVA, and treatment response was evaluated using major pathological response (MPR), defined as ≤10% residual viable tumor cells at the time of surgical resection.

External validation was performed using five independent LUAD cohorts (total n = 126), including GSE190266 (n = 56), GSE190265 (n = 14), GSE166449 (n = 22), GSE126044 (n = 7), and GSE135222 (n = 27). All cohorts consisted of advanced-stage LUAD patients treated predominantly with nivolumab in later lines of therapy, and the frequency of driver mutations remained consistently low (< 5%) across datasets.

Module 0 — Technical

Foundational analyses were performed using standardized pipelines across transcriptomic cohorts. Differential expression was conducted with *edgeR* (v3.40.2), applying a false discovery rate threshold of < 0.05 and effect-size filtering using $\log_2$ fold-change > 1. Survival analyses incorporated Kaplan–Meier estimation with log-rank testing, complemented by multivariable Cox proportional hazards models adjusted for sex, clinical stage, and line of therapy. Broader evidence synthesis involved a fixed-effect meta-analysis across 21 non-ICB LUAD cohorts, with between-study heterogeneity quantified using the I^2 statistic.

Correlation profiling used Spearman's rank correlation coefficients to assess relationships between the target gene and key immune-related markers, including *CD8A*, *GZMB*, *PRF1*, *PDCD1*, *HAVCR2*, *FOXP3*, and *CD274*. Pathway-level enrichment analyses were performed using *clusterProfiler* (v4.10.0) with Hallmark gene sets from MSigDB v7.5.1. Immune activity metrics—including the GEP, IFN-γ, and ICR scores—were derived using the *singscore* package (v1.20.0). Tumor microenvironment composition was estimated using *EPIC* deconvolution (v1.1.5) with its standard reference matrix.

Module A — Technical

All Module A analyses were performed on the GSE161537 cohort (n = 60 LUAD). Bulk RNA-seq data were filtered to remove genes expressed at < 1 TPM in more than half of the samples, $\log_2$(TPM+1) transformed, and standardized using median/MAD-based z-scores. Immune activation was quantified using the 27-gene HOT signature (CCL19, CCR2, CCR4, CCR5,

CD27, CD40LG, CD8A, CXCL10, CXCL11, CXCL13, CXCL9, CXCR3, CXCR6, FASLG, FGL2, GZMA, GZMH, IDO1, IFNG, IRF8, LAG3, LYZ, MS4A1, PDCD1, TBX21, TLR7, TLR8), computed by singscore. EPIC deconvolution provided immune fractions and compartment-adjusted expression, including an immune-adjusted BCL2A1 metric.

Models were trained using logistic regression (saga solver, class_weight = 'balanced') with a 10-fold outer loop and a 5-fold inner loop. Performance was evaluated using ROC AUC, PR-AUC, Brier score, and calibration slope/intercept. The classification threshold was fixed using the out-of-fold Youden index.

Pairwise DeLong tests compared AUCs between baseline and augmented models incorporating Line_bin and Stage_bin. Out-of-fold AUCs and 95% confidence intervals were obtained across the same 10 folds. Additional DeLong contrasts evaluated whether reduced or clinical-only models differed significantly from the full $BCL2A1^{+}CD274^{+}$ HOT model ($p < 0.05$ as significance threshold). Net Reclassification Improvement (NRI) was calculated for each augmented model relative to its baseline, using the baseline threshold; NRI was decomposed into event and non-event components to quantify gains in correct classification.

Module B — Technical

External Validation Framework

External validation employed a comprehensive leave-one-cohort-out (LOCO) cross-validation strategy using locked transcriptomic models from Module A. The macro-validation framework treated each cohort as an independent validation set to simulate real-world deployment scenarios across populations of varying sizes.

Multi-Cohort Validation Design

Validation was conducted across five LUAD cohorts (n = 126 total patients- GSE190266, n = 5 6; GSE190265, n = 14; GSE166449, n = 22; GSE126044, n = 7; GSE135222, n = 27). Performan ce metrics were calculated per cohort and averaged with equal weights to prevent large-cohort bi as.

Benchmark Signature Implementation

Four established signatures were implemented: TIDE (sum of T-cell dysfunction and exclusion components), TIS (geometric mean of 18-gene Merck signature), IPS (weighted average of four components with suppressor scores subtracted), and IFNG (arithmetic mean of interferon-gamma response genes). Signature scores were standardized within each cohort using z-scores.

Statistical Analysis

Primary endpoints included macro-averaged AUC with 95% confidence intervals calculated from cohort-level variation. Secondary metrics included precision-recall AUC, Brier score,

sensitivity, specificity, PPV, and NPV. Pairwise comparisons used paired t-tests on cohort-level AUCs.

Clinical Utility Assessment

Clinical utility evaluation included: (1) Decision curve analysis across probability thresholds 15%-50%, (2) NRI and IDI calculations compared to CD274 alone, and (3) Calibration analysis with intercept-only recalibration. Clinical decision thresholds were evaluated at six probability ranges: 15% (liberal), 20% (standard), 25% (moderate), 30% (conservative), 40% (restrictive), and 50% (high-risk).

This organization keeps the essential findings in the main text while providing comprehensive methodological details in the supplement.

Module C — Single-Cell Analysis Technical Implementation

Preprocessing and Quality Control: Single-cell RNA-seq data were preprocessed using scanpy v1.9.3, following standard protocols. Raw count matrices were normalized to 10,000 reads per cell, then log1p-transformed. Principal component analysis (PCA) was computed using the top 2,000 highly variable genes, and neighborhood graphs were constructed using the first 50 principal components. Leiden clustering was performed at resolution 0.6, followed by UMAP embedding for visualization.

$CD8^+$ T-Cell Isolation and Filtering: $CD8^+$ T-cells were identified and isolated based on expression criteria: $CD3D^+CD8A^+CD4^-$. Contaminating NK and NKT cells were excluded using markers $NKG7^+GZMB^+CD3^-$. Additional quality filters included a minimum of 200 genes per cell, a maximum mitochondrial gene expression of 20%, and removal of potential doublets.

Subtype Annotation and Differential Expression: Differential gene expression analysis was performed using the rank_genes_groups function with the Wilcoxon rank-sum test. Marker genes were identified using stringent criteria: $\log_2$ fold-change > 1, area under ROC curve > 0.7, and Benjamini-Hochberg adjusted p-value < 0.01. Subtype annotations were based on established lineage markers from the literature, with manual curation to ensure biological consistency.

Statistical Validation: Subtype abundance differences between responder groups were assessed using Fisher's exact test. BCL2A1 expression differences were evaluated using the Mann-Whitney U test. Robustness was validated through 1000× permutation tests of subtype proportions, with empirical p-values calculated from null distributions.

Cell-Cell Communication Network Analysis

BCL2A1 Expression Stratification: $CD8^+$ T-cell subsets were stratified by BCL2A1 expression using the 95th percentile threshold within each functional subset (memory, effector, proliferating, terminally differentiated, transitional, and exhausted). This approach ensured subset-specific thresholds while maintaining statistical power for downstream communication analysis.

Multi-Network Construction: Three complementary interaction networks were systematically constructed to capture different aspects of immune cell communication: (1) Intra-CD8 networks examining communication patterns between different $CD8^+$ T-cell functional subsets, (2) Inter-immune networks assessing $CD8^+$ T-cell interactions with other significant immune cell populations including B cells, dendritic cells, and NK cells, and (3) CD8-myeloid networks focusing specifically on antigen presentation and costimulatory pathways between $CD8^+$ T-cells and myeloid populations including macrophages, monocytes, and dendritic cell subsets.

Ligand-Receptor Database Integration and Statistical Framework: Communication analysis integrated curated ligand-receptor databases from both LIANA (Ligand-Receptor Analysis) and CellPhoneDB to maximize coverage of biologically relevant interactions. Statistical significance of interactions was determined using permutation-based testing (n = 1000), with interaction strength quantified as the geometric mean of ligand and receptor expression levels within communicating cell pairs. Pathway-level analysis focused on immunologically relevant signaling cascades, including macrophage migration inhibitory factor (MIF) signaling, HLA class I antigen presentation pathways, costimulatory molecule interactions (CD28-CD80/CD86, CD40-CD40LG), and immune checkpoint pathways. Multiple testing correction was applied using the Benjamini-Hochberg procedure, with a false discovery rate of 5%.

Module D — Technical

Bulk deconvolution was performed using EPIC v1.1.5 with seven $CD8^+$ T-cell subtype signatures: Trm, Tcm1, activated, effector-memory, Tex, proliferating, and terminally differentiated. Five reference matrices were constructed to probe cell-level and gene-level robustness. The FULL matrix served as the baseline. The POS and NEG matrices were derived from $BCL2A1^+$ and $BCL2A1^-$ $CD8^+$ subsets, respectively, testing whether BCL2A1 expression defines distinct underlying cell populations (cell-level validation). Two ablation matrices evaluated gene-level dependency: noB removed BCL2A1 from all subtype signatures, while dropNB removed BCL2A1 together with its highly correlated genes.

Robustness analyses included 1,000-iteration bootstrap resampling and Gaussian noise perturbation (±5%, 200 iterations). For each iteration, EPIC was rerun, and subtype estimates were compared with the original using Spearman correlation and mean absolute deviation. These complementary approaches assessed sensitivity to gene removal, sampling variation, and technical noise, providing a comprehensive evaluation of deconvolution stability.

Module E — Technical

To evaluate the robustness and portability of the Tri-axis signature across experimental platforms, we performed simulation-based stress testing using three commonly used transcriptomic technologies: HTG EdgeSeq, NanoString nCounter, and RT-qPCR. For each platform, synthetic measurement noise, dropout rates, and platform-specific biases were added directly to the discovery cohort expression matrix to mimic realistic assay conditions.

Platform simulation models

Three platform-specific noise models were implemented.
(1) HTG EdgeSeq simulation: Gaussian measurement error with mean 0 and SD 0.10 was added to all genes, together with a fixed 5% random dropout rate.
(2) NanoString simulation: Gaussian error N(0, 0.12) was combined with a 2% dropout rate and an additional gene-specific bias drawn from a uniform distribution U(–0.05, 0.05), reflecting probe-level hybridization variability.
(3) RT-qPCR simulation: This model incorporated Gaussian noise N(0, 0.08), 0.5% dropout, amplification efficiency variability drawn from U(0.85, 1.05), and batch effects implemented as six technical batches contributing 10% between-batch variance.

Negative control models

To ensure that the observed performance was attributable to the multi-gene signal rather than artifacts of simulation, we generated negative-control models applying the *same* simulation parameters to four simplified baselines:
CD274 alone, BCL2A1 alone, BCL2A1 + CD274, and the 27-gene HOT signature alone. These served as benchmarks to verify that Tri-axis performance did not arise from trivial or single-gene effects.

Bootstrap and confidence interval estimation

All platform-specific performance estimates were obtained through 2,000 bootstrap iterations with resampling of patients with replacement. Ninety-five percent confidence intervals were derived from the 2.5th and 97.5th percentiles of the bootstrap distributions.

Stress-test experiments

To examine the limits of model stability, a series of perturbation tests was performed:
noise amplitude was increased from 0% to 200% of baseline; dropout rates were varied from 0–15%; RT-qPCR batch variance was expanded from 0–20%; amplification efficiency was perturbed in the range ±5% to ±20%; and gene-set ablation was applied to the HOT signature in increments of 10–50%. These tests assessed whether prediction performance degraded gradually or exhibited failure thresholds under extreme distortion.

Agreement and reproducibility metrics

Cross-platform agreement between simulated and original expression-derived predictions was quantified using Deming regression with $\lambda = 1$ to account for measurement error in both axes, Lin's concordance correlation coefficient (CCC), and Bland–Altman analysis to evaluate systematic bias. Threshold stability was additionally assessed by examining deviations from the baseline (discovery) threshold within ±10 %.

Software

R v4.3.3: sva, singscore v1.20.0, msigdbr v7.5.1, clusterProfiler v4.10.0, pROC v1.18.5.

Python v3.9: scanpy v1.9.3, dca v0.3.4, coral-ml v1.0.1, scikit-learn v1.3.1.

Figure S1. Meta-analysis of BCL2A1 expression and prognosis in lung adenocarcinoma (LUAD). This meta-analysis assesses the prognostic significance of BCL2A1 expression in patients with LUAD who did not receive immune checkpoint blockade (ICB) therapy, including those treated with chemotherapy, targeted therapy, or no systemic treatment. High BCL2A1 expression was associated with poorer prognosis (HR: 1.07, 95% CI: 1.01–1.14, p = 0.0203), suggesting a potential pro-tumorigenic role in the absence of ICB. The lack of heterogeneity (I-squared = 0%, p = 0.4739) indicates consistent findings across independent studies, reinforcing the robustness of this association.

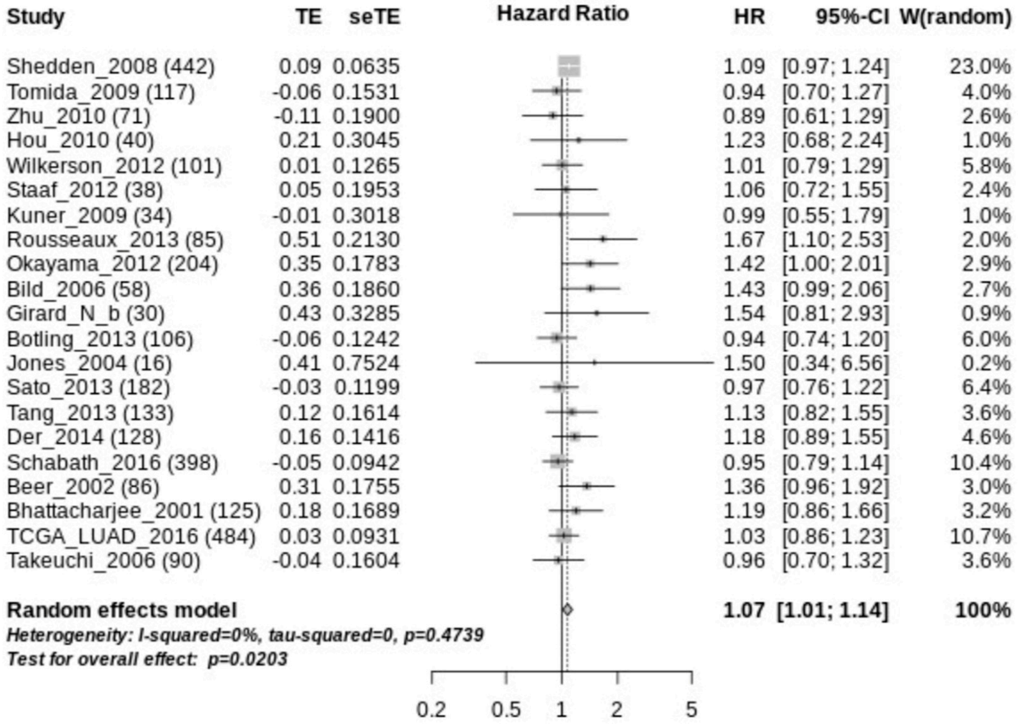

Figure S2. Comparative Evaluation of the Tri-axis Biomarker Against Established Transcriptomic Signatures. **(A)** presents the macro-averaged AUC ranking of five benchmark signatures. **(B)** shows cross-cohort classification performance across five independent LUAD immunotherapy datasets. (**C)** illustrates clinical utility metrics (NRI, IDI, and Net Benefit) benchmarked against the TIS baseline. (**D**) depicts the balance between model performance and gene-set complexity. (**E)** summarizes pairwise statistical comparisons between signatures using p-value heatmaps. (**F**) reports gene coverage rates across signatures relative to the whole transcriptome.

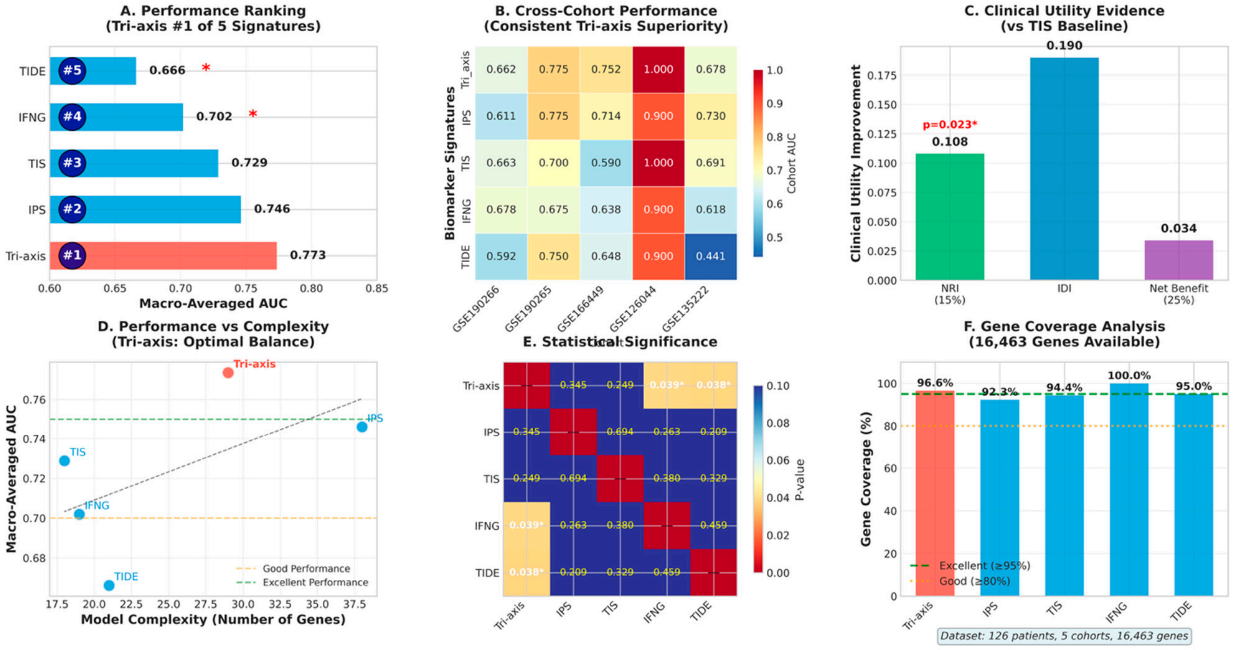

Figure S3. CD8$^+$ T-cell Communication Networks and Key Ligand–Receptor Interactions. **(A)** Statistical comparison of overall interaction strengths between BCL2A1-high and BCL2A1-low CD8+ T cells. Box plots show median and interquartile range. **(B)** Network of the top 20 interactions among CD8$^+$ T-cell subsets, represented as a force-directed graph. **(C)** Network of the top 20 interactions between CD8$^+$ T-cell subsets and other immune or stromal cell types. **(D)** Network of the top 20 interactions between CD8$^+$ T-cell subsets and myeloid populations. Node size represents connectivity; edge thickness represents interaction strength.

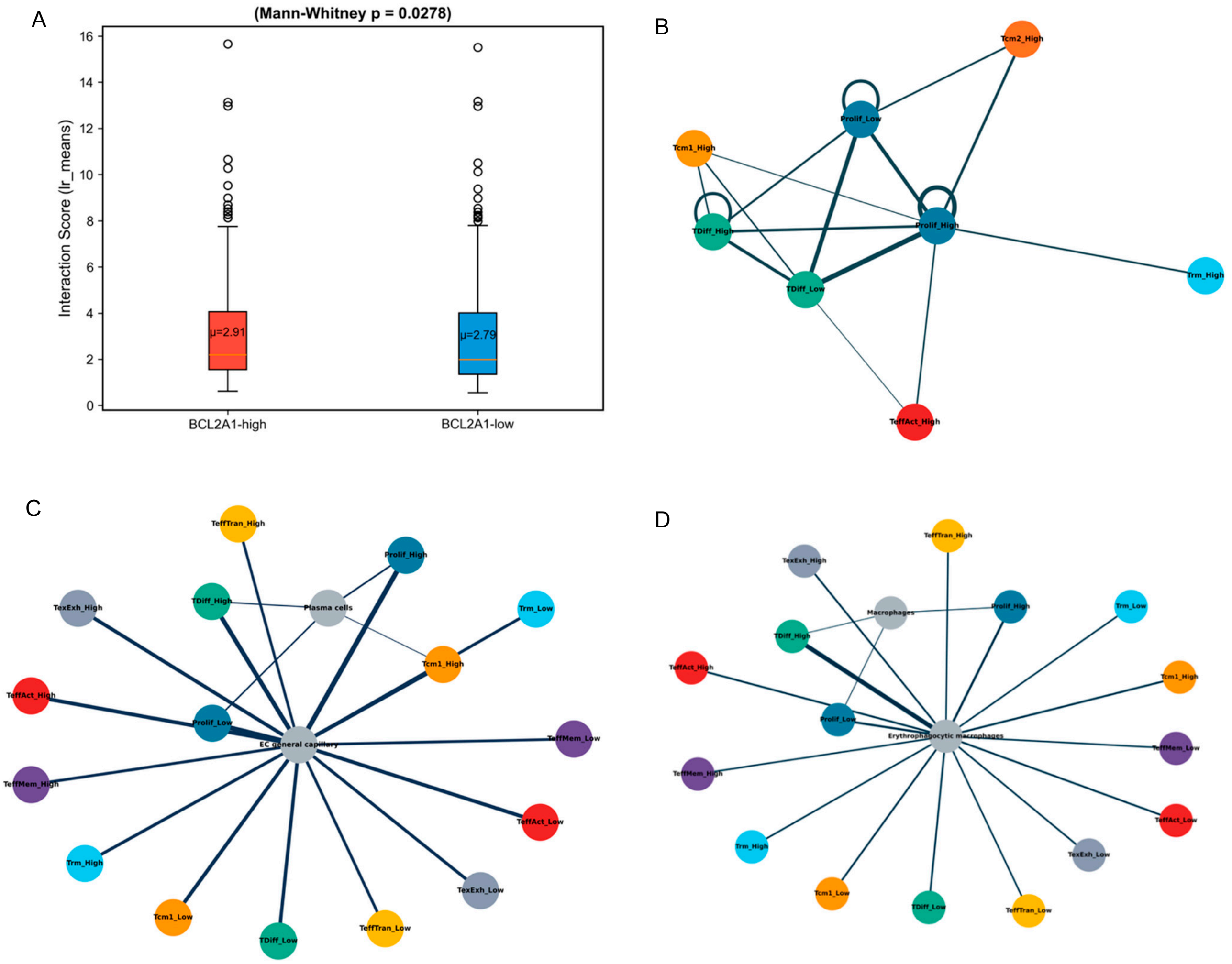

Figure S4. MIF signaling network heatmap. Mean interaction scores (lr_means) between source cell types (rows) and target cell types (columns) were displayed. BCL2A1-stratified CD8+ T cells are highlighted. Color intensity represents interaction strength. MIF exhibits the highest interaction scores among all pathways analyzed.

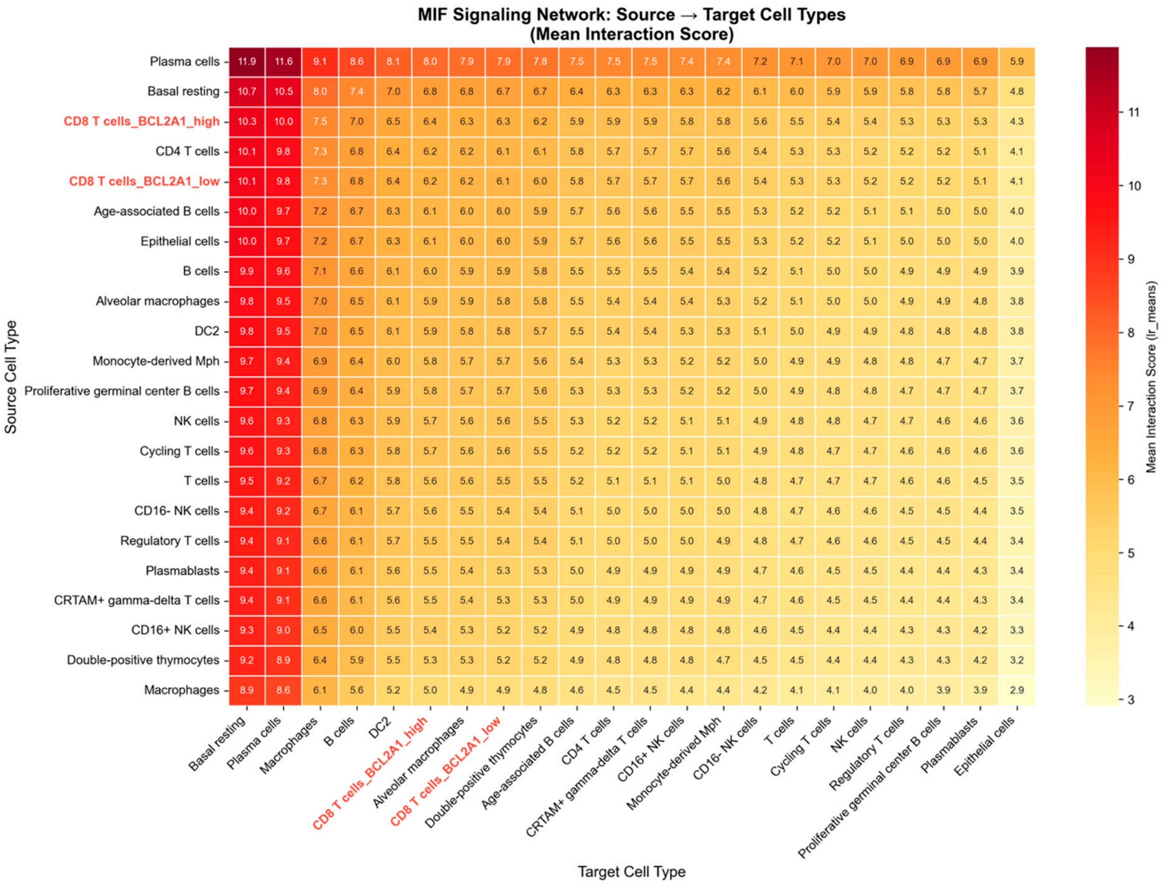

Table S1. Multivariable Logistic Regression of BCL2A1 Expression with ICB Response and Clinical Covariates. Logistic regression model examining the association between BCL2A1 expression and ICB response group (Responder vs Non-responder), adjusting for sex, clinical stage (early vs late), and treatment line (first-line vs later-line) in the discovery LUAD cohort (GSE161537; *n* = 60).

Variable	Coef	StdErr	t	p_value	CI_lower	CI_upper
Intercept	318.9198	168.2165	1.895889	0.063233	-18.1936	656.0332
Group[T.R]	315.8853	111.6086	2.830295	0.006481	92.21662	539.5539
sex[T.Male]	-40.5527	112.3071	-0.36109	0.719417	-265.621	184.5158
Stage_bin[T.late]	119.0746	157.8164	0.754513	0.45376	-197.197	435.3457
Line_bin[T.light]	-92.3485	107.4014	-0.85985	0.393607	-307.586	122.8886

Table S2. Correlation Between BCL2A1 Expression and Immune-Checkpoint or Effector Markers in the Discovery LUAD Cohort. Spearman correlation coefficients (ρ) and associated *p*-values between BCL2A1 and representative genes related to cytotoxic function (e.g., *PRF1*, *GZMB*, *IFNG*), $CD8^+$ T-cell identity (*CD8A*, *TBX21*), and immune checkpoints (*PDCD1*, *LAG3*, *CTLA4*, *HAVCR2*, *CD274*) in the ICB-treated LUAD cohort (GSE161537; *n* = 60).

Gene	Spearman_r	p_value
HAVCR2	0.796166	2.87E-14
PDCD1	0.687802	1.27E-09
PRF1	0.619561	1.31E-07
CD8A	0.600611	3.91E-07
CXCL13	0.568436	2.16E-06
LAG3	0.556488	3.89E-06
GZMB	0.555321	4.12E-06
TBX21	0.521423	1.94E-05
TNFRSF9	0.493748	6.1E-05
CTLA4	0.486635	8.06E-05
IFNG	0.482412	9.49E-05
FOXP3	0.408058	0.00121
CD274	0.12309	0.348778

Table S3. Correlation of BCL2A1 with Immune and Checkpoint Markers Stratified by Response Group. Spearman correlation coefficients between BCL2A1 expression and selected immune markers (including checkpoint genes and cytotoxic molecules), stratified by ICB response status (R = responder; NR = non-responder) in the discovery LUAD cohort (GSE161537).

Gene	Spearman_r	p_value	Group
HAVCR2	0.864615	1.23E-08	NR
CXCL13	0.766154	5.05E-06	NR
PDCD1	0.747692	1.13E-05	NR
CTLA4	0.716923	3.78E-05	NR
GZMB	0.667009	0.000198	NR
LAG3	0.661538	0.000233	NR
TNFRSF9	0.649231	0.000333	NR
CD8A	0.643077	0.000395	NR
TBX21	0.581538	0.001833	NR
IFNG	0.549402	0.003648	NR
FOXP3	0.539829	0.004421	NR
PRF1	0.532308	0.005122	NR
CD274	0.461197	0.01772	NR
HAVCR2	0.751261	3.06E-07	R
PRF1	0.604584	0.000152	R
PDCD1	0.552024	0.000713	R
CD8A	0.516272	0.001774	R
LAG3	0.502827	0.002439	R
IFNG	0.489687	0.003288	R
FOXP3	0.474408	0.004587	R
GZMB	0.429794	0.011185	R
CXCL13	0.426432	0.011907	R
TBX21	0.417876	0.013926	R
TNFRSF9	0.396486	0.020261	R
CTLA4	0.260199	0.137229	R
CD274	-0.09855	0.579243	R

Table S4. Out-of-fold performance metrics for baseline predictive models in the GSE161537 cohort. Models were trained using 10-fold cross-validation. Metrics reported include the area under the ROC curve (AUC_CV) with 95% confidence intervals, the precision-recall AUC (PR_AUC), and the Brier score. All models were evaluated on n = 60 patients.

Model	Features	AUC_CV	CI95	PR_AUC	Brier	n
**Whole expression (BCL2A1 + CD274 + hot)**	BCL2A1+CD274+hot	0.826	0.704-0.925	0.900	0.166	60
**Reduced (BCL2A1 + hot)**	BCL2A1+hot	0.822	0.698-0.929	0.886	0.164	60
**Full (ImmuneFraction + BCL2A1 + CD274 + hot)**	ImmuneFraction+BCL2A1+CD274+hot	0.818	0.697-0.919	0.889	0.170	60
**hot + CD274**	hot+CD274	0.756	0.621-0.877	0.808	0.181	60
**Immune expression (BCL2A1_Immune + CD274_Immune + hot)**	BCL2A1_Immune+CD274_Immune+hot	0.735	0.592-0.867	0.729	0.187	60
**hot only**	hot	0.727	0.575-0.864	0.729	0.179	60
**BCL2A1 + CD274**	BCL2A1+CD274	0.719	0.580-0.839	0.818	0.206	60
**BCL2A1 only**	BCL2A1	0.655	0.508-0.798	0.784	0.223	60
**CD274 only**	CD274	0.557	0.409-0.701	0.690	0.238	60
**ImmuneFraction only**	ImmuneFraction	0.468	0.317-0.623	0.569	0.253	60
**BCL2A1_Immune only**	BCL2A1_Immune	0.462	0.312-0.620	0.549	0.257	60
**CD274_Immune only**	CD274_Immune	0.447	0.297-0.597	0.551	0.251	60
**CD274_Immune + BCL2A1_Immune**	CD274_Immune+BCL2A1_Immune	0.414	0.275-0.562	0.510	0.260	60

Table S5. Pairwise DeLong test comparing AUCs between baseline models and the full model (BCL2A1 + CD274 + HOT). Each row reports the AUC of a baseline model (Model_A) versus the full model (Model_B), along with the associated DeLong p-value for testing the difference in discriminatory performance.

Model_A	Model_B	AUC_A	AUC_B	DeLong_p
CD274_Immune + BCL2A1_Immune	Whole expression (BCL2A1 + CD274 + hot)	0.414	0.826	3.289e-06
CD274_Immune only	Whole expression (BCL2A1 + CD274 + hot)	0.447	0.826	3.362e-05
BCL2A1_Immune only	Whole expression (BCL2A1 + CD274 + hot)	0.462	0.826	6.164e-05
ImmuneFraction only	Whole expression (BCL2A1 + CD274 + hot)	0.468	0.826	0.00022
CD274 only	Whole expression (BCL2A1 + CD274 + hot)	0.557	0.826	0.0020
BCL2A1 only	Whole expression (BCL2A1 + CD274 + hot)	0.655	0.826	0.0122
hot only	Whole expression (BCL2A1 + CD274 + hot)	0.727	0.826	0.063
Immune expression (BCL2A1_Immune + CD274_Immune + hot)	Whole expression (BCL2A1 + CD274 + hot)	0.735	0.826	0.085
BCL2A1 + CD274	Whole expression (BCL2A1 + CD274 + hot)	0.719	0.826	0.089
hot + CD274	Whole expression (BCL2A1 + CD274 + hot)	0.756	0.826	0.1839
Full (ImmuneFraction + BCL2A1 + CD274 + hot)	Whole expression (BCL2A1 + CD274 + hot)	0.818	0.826	0.6940
Reduced (BCL2A1 + hot)	Whole expression (BCL2A1 + CD274 + hot)	0.822	0.826	0.8860

Table S6. Out-of-fold AUC performance for augmented models including clinical covariates (GSE161537 cohort). Models were evaluated using 10-fold cross-validation. AUC_CV and 95% confidence intervals (CI95) are reported for each model. Clinical covariates included treatment line (Line_bin) and disease stage (Stage_bin), encoded as binary variables. All models were trained on n = 60 patients.

Model	Features	AUC_CV	CI95	n
**Whole expression (BCL2A1 + CD274 + hot)**	BCL2A1+CD274+hot	0.826	0.704-0.925	60
**Reduced (BCL2A1 + hot)**	BCL2A1+hot	0.822	0.698-0.929	60
**Reduced + Stage_bin**	BCL2A1+hot+Stage_bin_enc	0.822	0.698-0.929	60
**Whole + Line_bin + Stage_bin**	BCL2A1+CD274+hot+Line_bin_enc+Stage_bin_enc	0.822	0.698-0.927	60
**Reduced + Line_bin**	BCL2A1+hot+Line_bin_enc	0.821	0.697-0.929	60
**hot only**	hot	0.727	0.575-0.864	60
**BCL2A1 + Stage_bin**	BCL2A1+Stage_bin_enc	0.656	0.512-0.794	60
**BCL2A1 only**	BCL2A1	0.655	0.508-0.798	60
**BCL2A1 + Line_bin**	BCL2A1+Line_bin_enc	0.639	0.491-0.783	60
**CD274 only**	CD274	0.557	0.409-0.701	60
**Stage_bin only**	Stage_bin_enc	0.449	0.307-0.600	60
**Line_bin only**	Line_bin_enc	0.419	0.281-0.563	60

Table S7. DeLong test for pairwise AUC comparisons among augmented models (GSE161537 cohort). Each row reports the AUC difference and corresponding DeLong p-value for a given model contrast. "Incremental" comparisons test whether adding clinical covariates (e.g., Line_bin, Stage_bin) improves performance over expression-only models. "vs WHOLE" comparisons assess whether simplified or clinical-only models are significantly inferior to the full model (BCL2A1 + CD274 + HOT). Bolded rows ($p < 0.05$) indicate statistically significant differences.

**Contrast**	**Model_A**	**Model_B**	**AUC_A**	**AUC_B**	**DeLong_p**
**incremental**	BCL2A1 + Line_bin	BCL2A1 only	0.639	0.655	0.283
**incremental**	Whole + Line_bin + Stage_bin	Whole expression	0.822	0.826	0.719
**incremental**	Reduced + Line_bin	Reduced	0.821	0.822	0.727
**incremental**	BCL2A1 + Stage_bin	BCL2A1 only	0.656	0.655	0.957
**incremental**	Reduced + Stage_bin	Reduced	0.822	0.822	1.0
**vs WHOLE**	Line_bin only	Whole expression	0.419	0.826	1.848e-06
**vs WHOLE**	Stage_bin only	Whole expression	0.449	0.826	6.093e-05
**vs WHOLE**	CD274 only	Whole expression	0.557	0.826	0.002
**vs WHOLE**	BCL2A1 + Line_bin	Whole expression	0.639	0.826	0.0075
**vs WHOLE**	BCL2A1 only	Whole expression	0.655	0.826	0.0121
**vs WHOLE**	BCL2A1 + Stage_bin	Whole expression	0.656	0.826	0.013
**vs WHOLE**	hot only	Whole expression	0.727	0.826	0.063
**vs WHOLE**	Whole + Line_bin + Stage_bin	Whole expression	0.823	0.826	0.719
**vs WHOLE**	Reduced + Line_bin	Whole expression	0.821	0.826	0.840
**vs WHOLE**	Reduced (BCL2A1 + hot)	Whole expression	0.822	0.826	0.886
**vs WHOLE**	Reduced + Stage_bin	Whole expression	0.822	0.826	0.886

Table S8. Net Reclassification Improvement (NRI) for augmented models compared to baseline models (GSE161537 cohort). Each row compares an augmented model with its baseline using Net Reclassification Improvement (NRI), with classification thresholds derived from the baseline model's Youden index. NRI is decomposed into event (responders) and non-event (non-responders) contributions. Positive NRI_total indicates improved classification.

Pair	Baseline	Augmented	Baseline_threshold_Youden	NRI_events	NRI_nonevents	NRI_total	n_events	n_nonevents
**BCL2A1 + Line_bin vs BCL2A1 only**	BCL2A1 only	BCL2A1 + Line_bin	0.565	0.029	-0.038	-0.009	34	26
**BCL2A1 + Stage_bin vs BCL2A1 only**	BCL2A1 only	BCL2A1 + Stage_bin	0.565	-0.029	-0.038	-0.068	34	26
**Reduced + Line_bin vs Reduced (BCL2A1 + hot)**	Reduced (BCL2A1 + hot)	Reduced + Line_bin	0.488	0.0	0.0	0.0	34	26
**Reduced + Stage_bin vs Reduced (BCL2A1 + hot)**	Reduced (BCL2A1 + hot)	Reduced + Stage_bin	0.488	0.0	0.0	0.0	34	26
**Whole + Line_bin + Stage_bin vs Whole expression (BCL2A1 + CD274 + hot)**	Whole expression (BCL2A1 + CD274 + hot)	Whole + Line_bin + Stage_bin	0.757	-0.029	0.0	-0.029	34	26

Table S9. Macro discrimination metrics for models evaluated across LOCO multicohort external validation (n = 126). Metrics include AUC with 95% confidence intervals, PR-AUC, Brier score, and classification performance at the locked Youden threshold (sensitivity, specificity, PPV, NPV).

Model	AUC	CI95	PR_AUC	Brier	Sensitivity	Specificity	PPV	NPV	n
BCL2A1 + CD274 + HOT	0.774	0.6645-0.8831	0.626	0.246	0.859	0.513	0.424	0.899	126
BCL2A1 + CD274	0.77	0.6581-0.8818	0.611	0.253	0.925	0.465	0.42	0.944	126
BCL2A1 + HOT	0.76	0.6518-0.8680	0.612	0.248	0.846	0.483	0.405	0.891	126
CD274 + HOT	0.755	0.6670-0.8432	0.608	0.252	0.871	0.442	0.398	0.896	126
BCL2A1 only	0.759	0.6445-0.8733	0.6	0.265	0.912	0.365	0.379	0.923	126
CD274 only	0.706	0.5682-0.8435	0.617	0.283	0.975	0.118	0.316	0.75	126
HOT only	0.749	0.6681-0.8290	0.584	0.257	0.843	0.441	0.389	0.867	126

Table S10. Calibration and Brier score metrics before and after intercept-only recalibration (LOCO multicohort). Models were evaluated in raw form (original intercept) and after intercept-only recalibration ("POST") using the validation cohorts. Metrics include AUC, Brier score, calibration intercept and slope, and intercept shift.

Model	Type	AUC	Brier	Cal_intercept	Cal_slope	Intercept_shift
BCL2A1 + CD274 + HOT	RAW	0.774	0.2676	-1.214	0.514	
BCL2A1 + CD274 + HOT	POST	0.774	0.1946	-0.22	0.748	-1.343
BCL2A1 + CD274	RAW	0.77	0.2725	-1.147	0.576	
BCL2A1 + CD274	POST	0.77	0.2031	-0.561	0.351	-1.189
BCL2A1 + HOT	RAW	0.76	0.2674	-1.216	0.557	
BCL2A1 + HOT	POST	0.76	0.1963	-0.266	0.692	-1.324
CD274 + HOT	RAW	0.755	0.2669	-1.228	0.641	
CD274 + HOT	POST	0.755	0.1995	-0.39	0.545	-1.27
BCL2A1 only	RAW	0.759	0.2805	-1.128	0.571	
BCL2A1 only	POST	0.759	0.2054	-0.673	0.221	-1.121
CD274 only	RAW	0.706	0.2892	-1.087	0.527	
CD274 only	POST	0.706	0.2078	-0.834	0.037	-0.977
HOT only	RAW	0.749	0.2672	-1.219	0.681	
HOT only	POST	0.749	0.2013	-0.459	0.462	-1.237

Table S11. Net benefit from decision curve analysis after intercept-only recalibration (LOCO multicohort). Net benefit (NB) was computed for each model across a range of probability thresholds, and the maximum post-recalibration NB (Max_NB_Post) and corresponding optimal threshold are reported.

Model	Optimal _Thr	Max_NB _Pos	Clinical_Interp retation	NB_at_ 15%	NB_at_ 20%	NB_at_ 25%	NB_at_ 30%	NB_at_ 40%	NB_at_ 50%
BCL2A1 + CD274 + HOT	0.15	0.17708	15% (Liberal)	0.17708	0.15338	0.12919	0.09036	0.02814	0
BCL2A1 + CD274	0.15	0.16979	15% (Liberal)	0.16979	0.11791	0.05472	0.02542	0.00303	0
BCL2A1 + HOT	0.15	0.16821	15% (Liberal)	0.16821	0.13359	0.12205	0.08883	0.02208	0
CD274 + HOT	0.15	0.1727	15% (Liberal)	0.1727	0.12973	0.08174	0.05187	0.02814	0
BCL2A1 only	0.15	0.16979	15% (Liberal)	0.16979	0.11791	0.0591	0.01725	0	0
CD274 only	0.15	0.16979	15% (Liberal)	0.16979	0.11791	0.0591	0.00519	0	0
HOT only	0.15	0.16979	15% (Liberal)	0.16979	0.11976	0.06936	0.02255	0.02208	0

Table S12. Net Reclassification Improvement (NRI) and IDI for Benchmark Models Compared with CD274 Baseline. This table summarizes the NRI, NRI components (events and non-events), IDI, and reclassification directions for each model compared with the CD274-only baseline (N = 126). Positive NRI indicates improved overall reclassification, driven primarily by gains in non-events across models.

Model	vs_Baseline	NRI	NRI_Events	NRI_NonEvents	IDI	Events_Up	Events_Down	NonEvents_Up	NonEvents_Down	N
BCL2A1 + CD274 + HOT	CD274 only	0.218	-0.108	0.326	0.185	0	4	0	29	126
BCL2A1 + HOT	CD274 only	0.206	-0.108	0.315	0.168	0	4	0	28	126
CD274 + HOT	CD274 only	0.186	-0.027	0.213	0.123	0	1	0	19	126
HOT only	CD274 only	0.056	0	0.056	0.1	0	0	0	5	126
BCL2A1 + CD274	CD274 only	0.029	-0.027	0.056	0.087	0	1	0	5	126
BCL2A1 only	CD274 only	0.034	0	0.034	0.056	0	0	0	3	126
CD274 only	Reference	0	0	0	0	0	0	0	0	126

Table S13. Permutation test results comparing CD8+ T-cell subtype frequencies between MPR and non-MPR groups (GSE176021). Observed differences (Δ) in subtype proportions were tested using 1,000 random label permutations. Reported values include the observed difference in mean frequency (Observed_Delta), empirical permutation p-value (p_perm), 95% null distribution confidence intervals (CI_Lower_95, CI_Upper_95), and FDR-adjusted p-values.

Subtype	Observed_Delta	p_perm	CI_Lower_95	CI_Upper_95	fdr
CD8_Tcm_1	0.048736	0.000999	-0.00318	0.003322	0.000999
CD8_Teff_Activated	0.161587	0.000999	-0.00445	0.003929	0.000999
CD8_Tcm_2	-0.00924	0.000999	-0.0025	0.002404	0.000999
CD8_Proliferating	0.134895	0.000999	-0.00341	0.003335	0.000999
CD8_Tex_Exhausted	-0.29743	0.000999	-0.00396	0.003809	0.000999
CD8_Teff_Memory	-0.14141	0.000999	-0.00279	0.002849	0.000999
CD8_Teff_Transitional	-0.09082	0.000999	-0.00409	0.00377	0.000999
CD8_Trm	0.219743	0.000999	-0.00373	0.00373	0.000999
CD8_Terminally_Differentiated	-0.02606	0.000999	-0.0012	0.001234	0.000999

Table S14. Summary of CD8$^+$ subtype enrichment in responders vs non-responders across POS, NEG, and FULL reference matrices (GSE161537). For each reference, mean subtype abundance in responders (Mean_R) and non-responders (Mean_NR) is reported, along with the absolute difference (Delta), Mann–Whitney U test q-value (MW_padj), and Fisher's exact test q-value (Fisher_padj).

Reference	Subtype	Mean_R	Mean_NR	Delta	MW_padj	Fisher_padj
POS	CD8_Trm	118,020	52,900	65,120	0,751	0,929
POS	CD8_Teff_Memory	103,341	76,268	27,073	0,751	0,929
POS	CD8_Proliferating	289,051	211,901	77,150	0,751	0,929
POS	CD8_Tex_Exhausted	0,276	3,656	-3,380	0,751	0,929
POS	CD8_Tcm_1	7,310	3,864	3,447	0,751	0,929
POS	CD8_Teff_Activated	44,808	13,175	31,633	0,751	0,929
POS	CD8_Terminally_Differentiated	61,972	22,370	39,602	0,751	1
NEG	CD8_Tcm_1	6,009	4,930	1,079	0,806	0,724
NEG	CD8_Terminally_Differentiated	58,437	25,504	32,933	0,550	0,724
NEG	CD8_Tex_Exhausted	1,266	5,090	-3,824	0,815	0,724
NEG	CD8_Trm	121,363	53,416	67,947	0,550	0,724
NEG	CD8_Proliferating	322,833	206,480	116,353	0,550	0,898
NEG	CD8_Teff_Memory	83,486	68,743	14,743	0,806	0,898
NEG	CD8_Teff_Activated	31,203	19,745	11,458	0,890	1
FULL	CD8_Teff_Memory	152,973	77,778	75,195	0,02	0,392
FULL	CD8_Trm	163,410	78,773	84,637	0,167	0,438
FULL	CD8_Tcm_1	33,283	4,453	28,830	0,02	0,438
FULL	CD8_Teff_Activated	27,114	17,995	9,119	0,900	0,933
FULL	CD8_Tex_Exhausted	9,751	7,940	1,8119	0,900	0,933
FULL	CD8_Terminally_Differentiated	22,055	12,710	9,345	0,900	0,933
FULL	CD8_Proliferating	217,394	184,890	32,504	0,900	1

Table S15. Robustness of CD8⁺ Subtype Proportions to Marker Ablation. Spearman correlation coefficients (r) and corresponding p-values comparing CD8⁺ subtype proportion estimates between the full reference matrix (FULL) and two ablation variants: noB (excluding BCL2A1) and dropNB (excluding BCL2A1 + its correlated genes). High correlations (e.g., r > 0.60) with significant p-values indicate stable subtype estimation despite marker removal.

Subtype	r_FULL_vs_noB	p_FULL_vs_noB	r_FULL_vs_dropNB	p_FULL_vs_dropNB
CD8_Teff_Activated	0.69	0.0000	0.28	0.02770
CD8_Tcm_1	0.33	0.0109	0.27	0.03681
CD8_Trm	0.91	0.0000	0.60	0.00000
CD8_Proliferating	0.93	0.0000	0.38	0.00266
CD8_Tex_Exhausted	0.26	0.0485	0.07	0.61388
CD8_Teff_Memory	0.67	0.0000	0.22	0.08477
CD8_Terminally_Differentiated	0.73	0.0000	0.37	0.00389

Table S16. Predictive performance of logistic regression models using ablated CD8$^+$ subtype signatures (GSE161537). AUCs were computed for multivariate models using the FULL, noB (excluding BCL2A1), and dropNB (excluding BCL2A1 + correlated genes) reference matrices. DeLong p-values compare each ablated model to the FULL signature.

Subtype	AUC_FULL	AUC_noB	AUC_dropNB	p_noB_vs_FULL	p_dropNB_vs_FULL
CD8_Teff_Activated	0.688	0.714	0.721	0.962	0.951
CD8_Tcm_1	0.688	0.714	0.721	0.962	0.951
CD8_Trm	0.688	0.714	0.721	0.962	0.951
CD8_Proliferating	0.688	0.714	0.721	0.962	0.951
CD8_Tex_Exhausted	0.688	0.714	0.721	0.962	0.951
CD8_Teff_Memory	0.688	0.714	0.721	0.962	0.951
CD8_Terminally_Differentiated	0.688	0.714	0.721	0.962	0.951

Table S17. Exclusive and shared top-ranking genes from POS and NEG CD8^{+} reference signatures (GSE161537). Genes were classified as POS-only, NEG-only, or Shared based on their inclusion among the top markers in each BCL2A1-stratified reference matrix.

Gene Group	Gene
POS-only	BCL2A1
POS-only	IER2
POS-only	NR4A2
POS-only	SOD1
POS-only	YWHAB
NEG-only	AKAP13
NEG-only	C12orf57
NEG-only	EVL
NEG-only	HLA-DPB1
NEG-only	HNRNPDL
NEG-only	RNF213
NEG-only	STK17A
NEG-only	SYTL3
NEG-only	TUBB4B
NEG-only	TXNIP
Shared	ACTB
Shared	ACTG1
Shared	ADGRE5
Shared	ALOX5AP
Shared	ANXA1
Shared	ARHGDIB
Shared	ARPC2
Shared	ATP1B3
Shared	ATP5F1E
Shared	ATP5MC2

Table S18. Stability of CD8+ Subtype Estimates Under Bootstrap and Noise Perturbation. Quantification of robustness for CD8+ subtype deconvolution across three reference matrices (FULL, POS, NEG), assessed via 1,000× bootstrap resampling and ±5% Gaussian noise injection. For each subtype, the interquartile range (Q25–Q75), standard deviation (SD), and mean absolute difference (Diff) from baseline estimates were calculated. Low SDs and minimal Diff values (< 0.0026) indicate high stability of subtype estimates across perturbation conditions.

group	subtype	Q25	Q75	SD	Diff
FULL	CD8_Teff_Activated	7.04129108	7.36405877	0.25254984	0.00256315
FULL	CD8_Tcm_1	6.41510995	6.69529742	0.22682748	0.00229313
FULL	CD8_Trm	6.97633802	7.3010558	0.25071059	0.00258526
FULL	CD8_Proliferating	5.88656797	6.14794849	0.20602754	0.00240341
FULL	CD8_Tex_Exhausted	5.7095862	5.96016632	0.19693686	0.00221717
FULL	CD8_Teff_Memory	5.79011289	6.08051953	0.2140274	0.00250416
FULL	CD8_Terminally_Differentiated	6.87602799	7.22492538	0.25616874	0.00232908
POS	CD8_Teff_Activated	7.04061075	7.34951078	0.24709004	0.00251497
POS	CD8_Tcm_1	6.42564652	6.719208	0.22300486	0.00238253
POS	CD8_Trm	6.97525206	7.28472225	0.24327017	0.00228454
POS	CD8_Proliferating	5.86057881	6.12087937	0.20164886	0.00233266
POS	CD8_Tex_Exhausted	5.77065728	6.03265499	0.19593235	0.00244454
POS	CD8_Teff_Memory	5.87920461	6.14413275	0.20897618	0.00258383
POS	CD8_Terminally_Differentiated	6.78729189	7.10220127	0.24493218	0.00239803
NEG	CD8_Teff_Activated	7.00846675	7.36949348	0.26346638	0.00239207
NEG	CD8_Tcm_1	6.38796376	6.71023101	0.23531962	0.00245679
NEG	CD8_Trm	6.93617817	7.30570188	0.26165795	0.00229828
NEG	CD8_Proliferating	5.87122344	6.16496398	0.21542777	0.00241968
NEG	CD8_Tex_Exhausted	5.67801746	5.96028609	0.20395821	0.00228062
NEG	CD8_Teff_Memory	5.78245274	6.08074682	0.22109557	0.00223316
NEG	CD8_Terminally_Differentiated	6.87863812	7.23889428	0.26514519	0.00227287

Table S19. Cross-Platform Concordance of the Tri-Axis Signature. Summary of *in silico* simulations assessing Tri-Axis reproducibility across HTG EdgeSeq, NanoString nCounter, and RT-qPCR. Shown are CV, dropout, overall Spearman ρ (95 % CI, 2,000 bootstraps), Cohen's κ, and gene-level correlations for BCL2A1 and CD274. All platforms met predefined acceptance ($\rho \geq 0.90$, $\kappa \geq 0.75$).

Platform	CV	Dropout	ρ (95% CI)	κ	BCL2A1 ρ	CD274 ρ
HTG	10%	5%	0.986 (0.976-0.997)	0.933	0.955	0.957
NanoString	12%	2%	0.993 (0.976-0.997)	0.933	0.990	0.960
RT-qPCR	8%	0.5%	0.982 (0.960-0.989)	0.867	0.975	0.976

Table S20. Negative-Control Signature Performance Across Platforms. *In silico* benchmarking of negative-control models—BCL2A1 alone, CD274 alone, and HOT-only—against the Tri-Axis reference across three targeted platforms. Reported metrics include Spearman ρ, Cohen's κ, and bootstrap 95 % CI (2,000 iterations). These controls validate the specificity of Tri-Axis robustness by distinguishing true biological stability from random correlation.

Platform	Signature	ρ	κ	Bootstrap ρ CI
HTG	Tri-Axis	0.986	0.933	0.976-0.997
	BCL2A1	0.955	0.933	0.777-0.970
	CD274	0.957	0.667	0.949-0.993
	HOT	0.997	0.967	0.987-0.998
NanoString	Tri-Axis	0.993	0.933	0.976-0.997
	BCL2A1	0.990	0.900	0.942-0.993
	CD274	0.960	0.767	0.959-0.995
	HOT	0.998	0.933	0.991-0.999
RT-qPCR	Tri-Axis	0.982	0.867	0.960-0.989
	BCL2A1	0.975	0.867	0.939-0.990
	CD274	0.976	0.900	0.943-0.987
	HOT	0.987	0.900	0.969-0.992

Table S21. Stress-Test Simulation of Platform Robustness. Results of *in silico* stress tests evaluating Tri-Axis stability under increasing experimental perturbations. Simulated conditions included elevated noise (30 % CV), dropout (15 %), RT-qPCR batch variance (0–20 %), amplification efficiency deviation (± 20 %), and HOT-signature ablation (50 %). Reported values are platform-specific Spearman ρ after each perturbation.

Test	Condition	HTG ρ	NanoString ρ	RT-qPCR ρ
Noise	30% CV	0.951	0.977	N/A
Dropout	15%	0.974	0.981	N/A
Batch	0%	N/A	N/A	0.994
	10%	N/A	N/A	0.982
	20%	N/A	N/A	0.938
Amp eff	±20%	N/A	N/A	0.995
Ablation	50% HOT	0.990	0.991	0.979

Table S22. Agreement Analysis and Response-Stratified Performance. Summary of *in silico* agreement and subgroup analyses assessing Tri-Axis reproducibility across patient response categories. Metrics include Lin's concordance correlation coefficient (CCC), Bland–Altman bias, and paired Spearman ρ / Cohen's κ values for all samples, responders, and non-responders.

**Platform**	**Lin's CCC**	**Bias**	**All (n = 60)**	**Responders (n = 34)**	**Non-Resp (n = 26)**
			ρ / κ	ρ / κ	ρ / κ
HTG	0.986	-0.001	0.986/0.933	0.988/0.816	0.987/1.000
NanoString	0.989	-0.001	0.993/0.933	0.989/0.900	0.991/0.933
RT-qPCR	0.982	-0.001	0.982/0.867	0.984/0.867	0.980/0.867
